# Exploring the impact of pretreatment and particle size variation on properties of rubberized concrete

**DOI:** 10.1038/s41598-025-96402-y

**Published:** 2025-04-03

**Authors:** Dhiraj Agrawal, Khalid Ansari, Uday Waghe, Manmohan Goel, S. P. Raut, Harshal Warade, Essam Althaqafi, Saiful Islam, Osamah J. Al-sareji

**Affiliations:** 1 Department of Civil Engineering, Yeshwantrao Chavan College of Engineering (YCCE), Nagpur, 441110 India; 2 Department of Applied Mechanics, Visvesvaraya National Institute of Technology (VNIT), Nagpur, 440010 India; 3 Civil Engineering Department, College of Engineering, King Khalid University, 61421 Abha, Saudi Arabia; 4 Sustainability Solutions Research Lab, Faculty of Engineering, University of Pannonia, Egyetem Str. 10, Veszprém, 8200 Hungary

**Keywords:** Rubber particles, Rubberized concrete, Strength, Pre-treatment of rubber particles, Abrasion resistance, Varying sizes of waste tire rubber, Engineering, Civil engineering

## Abstract

This study comprises the influence of particle sizes of discarded tire rubber with and without pre-treatment to check the hardened properties of rubberized concrete (RC) in analogy to control concrete. Fine aggregates from concrete are volumetrically substituted using untreated and pre-treated rubber particles of three different sizes up to 20% in part, with an increment of 5%. Pre-treatment using two different pre-treatment methods i.e., sodium hydroxide (NaOH) and silica fume and NaOH was adopted. Properties of fresh concrete-i.e., density, workability, and toughened concrete strengths as compressive, flexural, and indirect tensile strengths were assessed with the control concrete. Along with these, unconventional properties like cylindrical compressive strength and abrasion, resistance to impact, and water absorption are compared. The mechanical properties of RC were found to be similar to conventional concrete, as the compressive strength for RC with pretreated rubber fibers up to 10% obtained was 61.5 MPa while flexural and split tensile strength was above 5.6 MPa. The abrasion resistance was obtained for RC with rubber fibers from 0.98 to 1.46 mm up to 20% substitution against 1.34 mm of control concrete. Modified concrete with pre-treated rubber particles showed better performance than concrete with untreated rubber particles with coarser sizes.

## Introduction

Due to hasty progress in automobile industries and the enormous usage of road transport vehicles, the problem associated with discarded tires has increased. In recent years, according to a research study^1^, 5000 million tires will be discarded by 2030 consistently through the year. These discarded tires are mostly stockpiled and a meager of these are recycled. This activity raised a concern for our outdoor environment as these stockpiled end-of-life tires (ELT) pollute the land, and water bodies, and spread diseases, burning of such ELT commonly known as pyrolysis, is a common, inexpensive, and easiest process mostly used in South-East Asia causes the grievous problem of air pollution^2,3^. To overcome these problems most nations banned the processes of landfilling using discarded tires, and burning/pyrolysis^4^. Alternate options are being invented and implemented for recycling ELT up to the possible quantity from the past three decades in various sectors. At the same time, undeviating evolution is observed in the infrastructure and construction industries in the last 30–40 years^5^. This also caused an increase in demand for elements of concrete. The construction industry is realizing an acute shortage of elements of concrete and mortar, there is a prodigious necessity to discover alternatives for fine aggregates, coarse aggregates, and cement. Extensive research has been carried out for finding a partial or complete alternative to cement and researchers have come out with promising results^6–8^. On the contrary, limited research was conducted for inventing a substitute for sand even though the scarcity of sand is increasing day by day around the globe with damage to aquatic life due to its extraction from rivers. The use of ELT as a fractional/ complete switch for aggregates has been inspected by many authors to make concrete more flexible^9^. Some studies on part or complete substitution of aggregates to make the concrete sustainable and to minimize the impairment of the environment by ELT are reviewed before conducting this investigation study.

Rubber tire scraps combined with other materials to partially replace sand in RC concrete have an impact on the modified concrete, which has shown a diminution in compressive strength as the rubber increases^10^. The indirect tensile strength and modulus of elasticity are reported to be decreased by 22% after a 20% exchange of sand using rubber particles, while the fracture energy is amplified by 2 and 3 times for a 10 and 20% swap of sand by discarded rubber particles^11^. Another research^12^ was conducted to recognize the compressive strength and noted that compressive strength is weakened by 30% at 20% substitution of sand. Along with this, they also carried out detailed research on RC columns under seismic loadings and concluded that the viscous damping is decreased for RC columns in concerning to normal concrete. The ductility of RC is boosted by the insertion of rubber particles, while the compressive strength decreases owing to weak ties among rubber and cement matrix^13^. In another research work on the effect of an unfriendly environment on RC, the reduction in strength and loss of weight are more in the control concrete than in RC. The water absorption for RC is observed to be higher. The carbonation resistance is similar to control concrete with up to 12.5% substitution of sand by rubber particles^14^. Agrawal et al.^15^ experimented with the application of RF in concrete and concluded that the optimal drop in strengths occurred for untreated RF while utilizing pretreated RF in concrete achieved satisfactory results up to 10% replacement. Also, the experimental work^16^ presented that, the unit weight, slump value, compressive strength, and elastic modulus are degraded due to an augmentation in rubber content. The decline of 48.3% in compressive strength for a 50% substitution of sand was observed. In the experimental work ^17^, the RF is utilized as a partial switch of sand up to 25% with an increment of 5% and it is observed that increased RF doses result in a loss in compressive strength. Authors^18^ performed a research study on RC with varying water-cement (w/c) ratios and commented that the slump and compressive strength are reduced with an augmentation in the percentage of rubber particles. The amalgamation of SF in concrete with RF showed improvement in compressive strength. Su et al.^19^ concluded from their study on RC with changing rubber particle sizes that the compressive strength is lowered for all sizes of rubber particles when used separately and the decrement ratio for all the cases is around 10% for 20% part replacement of sand using rubber particles. The rise in permeability is observed by incorporating rubber into concrete. Permeability is increased for modified concrete through the usage of bigger rubber particles is higher, in some cases, permeability is enhanced up to 200%^18–20^. According to various studies carried out, the indirect tensile and flexural strength of RC has shown almost a decline trend as linked to the control concrete after the alternative used for sand as CR or rubber particles. The cutback proportion for these tests is higher in the RC with bigger-size rubber particles. The average decrease in split tensile strength is recorded around 30% after a 30% substitute of sand by rubber particles^11,19,21–24^. Few researchers acknowledged the insertion of rubber particles enhanced the split tensile strength. The strength is enhanced by 4% for 20% fractional substitution of sand by rubber^13^. Uncommonly, some research works are also conducted on the pretreatment of rubber particles before their inclusion in the concrete. Emulsified asphalt, sodium hydroxide (NaOH), hydrochloric acid (HCl), and water-soaking methods are adopted for the treatment of rubber particles to make them rough and porous, and therefore proper bonding must be assumed between rubber particles and cement paste^13,21,22,25^. Tayeh^26^ demonstrated from research work conducted on the beam that the bending load, inertial load, and, impact tup are improved for modified concrete with the limited switch of sand by rubber. A study performed by Khalil et al.^27^ on self-compacting concrete (SCC) using CR determined the impact resistance and found an increased impact resistance for RC by the deployment of CR as an alternative for sand in SCC. Pre-treatment of rubber needs to be practiced in detail as limited research is carried out on this, pre-treatment of the varying particle sizes of rubber also showed variation in results, which cn also be a notable grey area in inventing sustainable concrete prepared using ELT. Xue and Shinozuka^28^ used SF on a trial basis to improve the strength of concrete with rubber particles and observed that compressive strength is condensed by 47% after 20% fractional switch of coarse aggregate by rubber particles with no SF, wherein the addition of SF decreased the degree of reduction for compressive strength. The damping ratio is extracted from the free vibration test on normal concrete and RC. The insertion of rubber improved the damping ratio by 62% and it showed that concrete containing rubber is more proficient to dissipate kinetic energy. Ossola and Wojcik^29^ conducted a study on cementitious composites with rubber particles using the ultra-violet (UV) radiation method for pretreatment. They also concluded that the strength losses due to the inclusion of rubber in cementitious composites were reduced due to the UV radiation process. Also,^30^ performed a study and concluded that the pre-coating of rubber particles has shown promising enrichment in the interfacial bonding of rubber particles and siliceous material when linked to the concrete with rubber without any pre-treatment, they detected growth in indirect tensile and compressive strength through 19% and 37% for concrete with pretreatment of CR. Authors^31^ carried out detailed research on the thermal treatment of CR. Heating time, particle size, and rubber content are variables for different mixes of RC tested in their study. Research^32^ concluded that by partially replacing the cement with SF and pretreating the rubber particles with NaOH, the mechanical characteristics of RC are enhanced.

All the reviewed research works have used single size of rubber particles and most of the experiments are conducted on untreated rubber particles, no outcome is derived on the utilization of diverse sizes of rubber particles and the outcome of pre-treatment of discarded rubber particles before its utilization in concrete as a partial or complete swap for aggregates. The variation in pre-treatment using different emulsifying agents like NaOH, HCl, etc. has not been studied. Along with this, the pretreatment method with immersion in NaOH and forming a coating using SF around pretreated rubber particles before the dry mixing process of concrete constituents is not studied.

In the current experimental research, a thorough examination was conducted to evaluate and compare the properties of fresh concrete, mechanical, and durability properties of RC with control concrete. The study has been taken on by switching sand up to 20% by rubber particles with an increment ratio of 5%, and by considering varying particle sizes of rubber as S-1 of size 300 microns in powdered form, S-2 of size 1.18 mm to 300 microns as CR, S-3 of size 2.36–1.18 mm as RF with aspect ratio up to 20. Two different pre-treatments are given to all sizes of rubber particles. Concrete’s slump was kept constant at 120 mm using controlled dosages of super-plasticizer.

## Experimental details

A total of 37 concrete mixes incorporating various types of rubber particles were made and examined for both fresh and hardened concrete parameters. The mix design was performed according to the guidelines set by Indian Standards^33^. The samples were cast in compliance with Indian Standards^34–36^, and they were tested and matched to control concrete as per American and Indian standards^34–39^.

### Materials and methodology

**Fig. 1:**
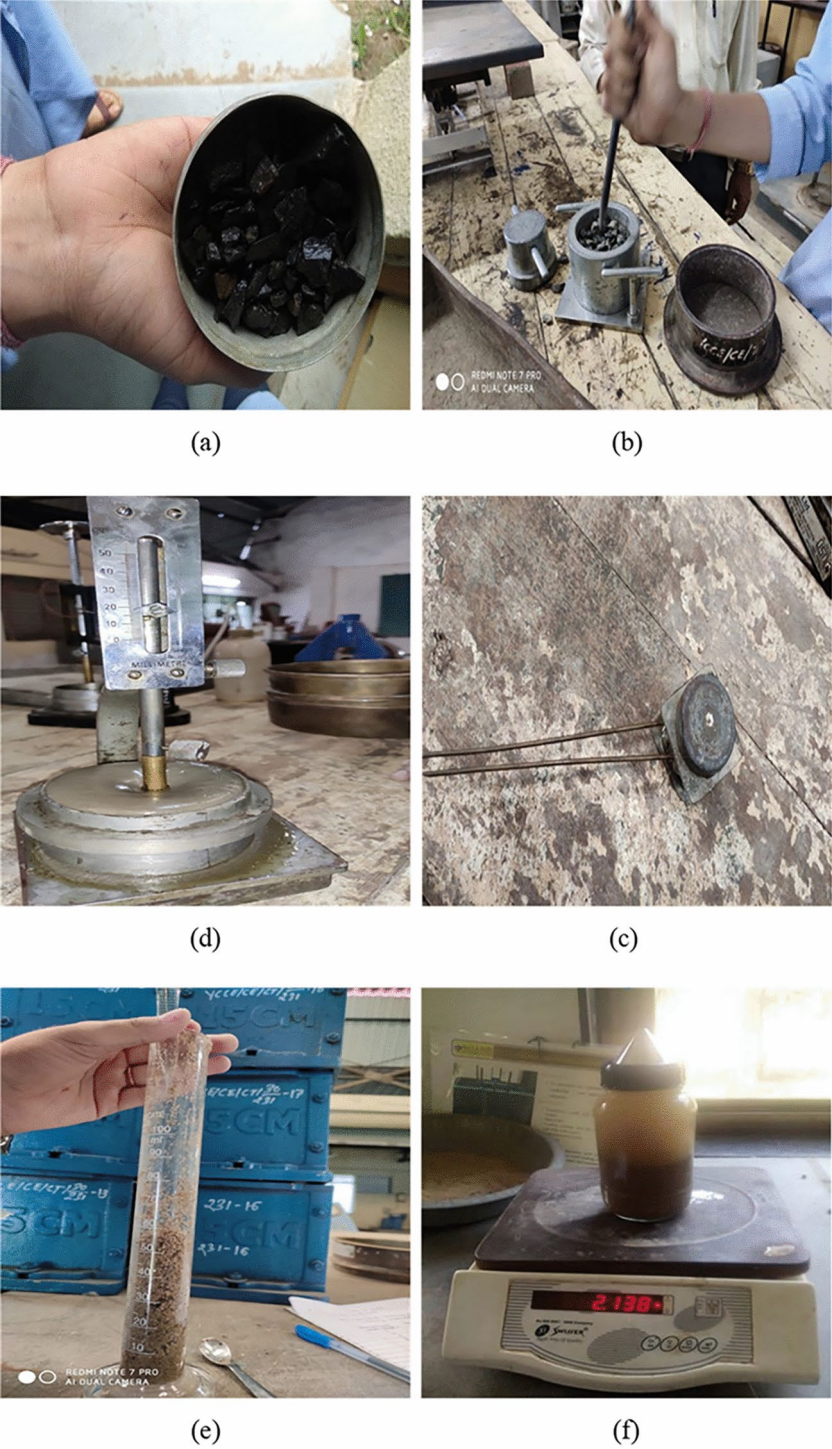
Tests on Aggregates: (**a**) Water Absorption of Coarse Aggregate, (**b**) Crushing Strength of Coarse Aggregate, (**c**) Soundness Test on Cement, (**d**) Consistency Test on Cement, (**e**) Bulking of Sand, and (**f**) Specific Gravity of Sand.

**Fig. 2:**
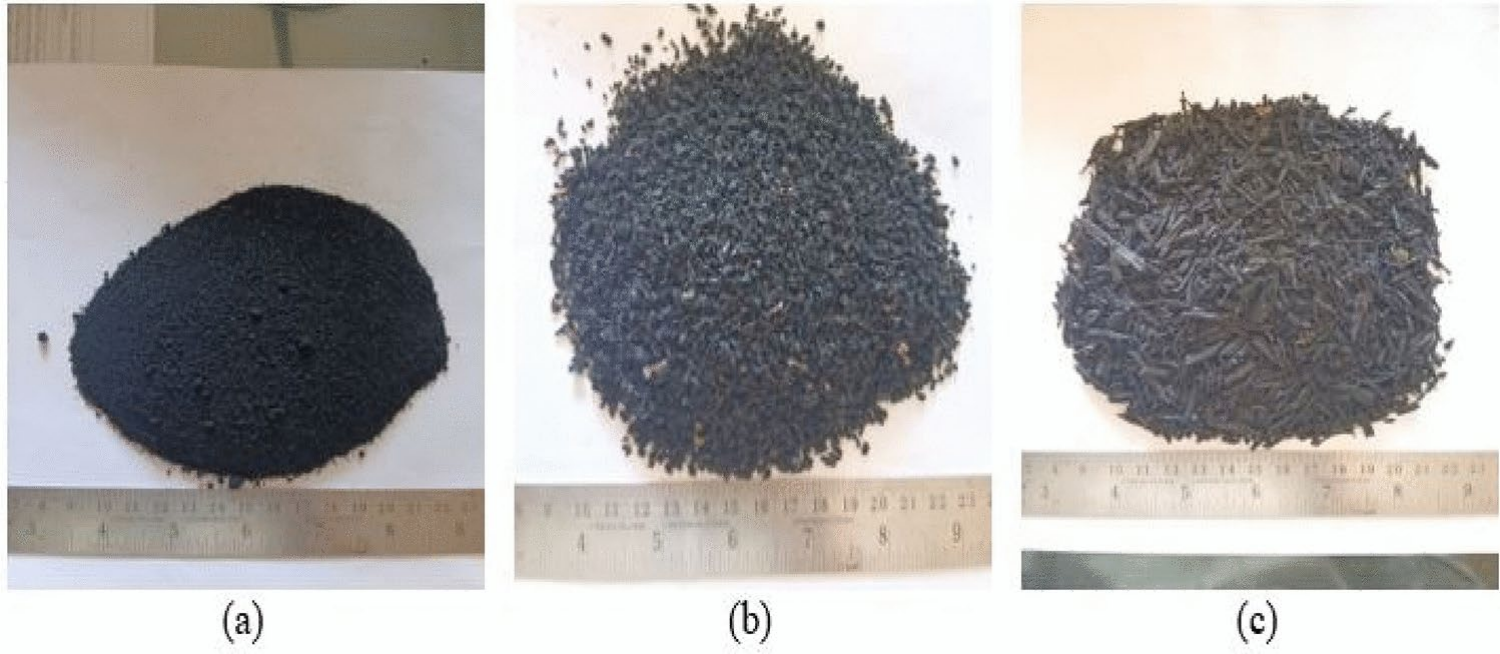
Rubber Particles: (**a**) Rubber Powder, (**b**) Crumb Rubber, and (**c**) Rubber Fiber.

**Fig. 3:**
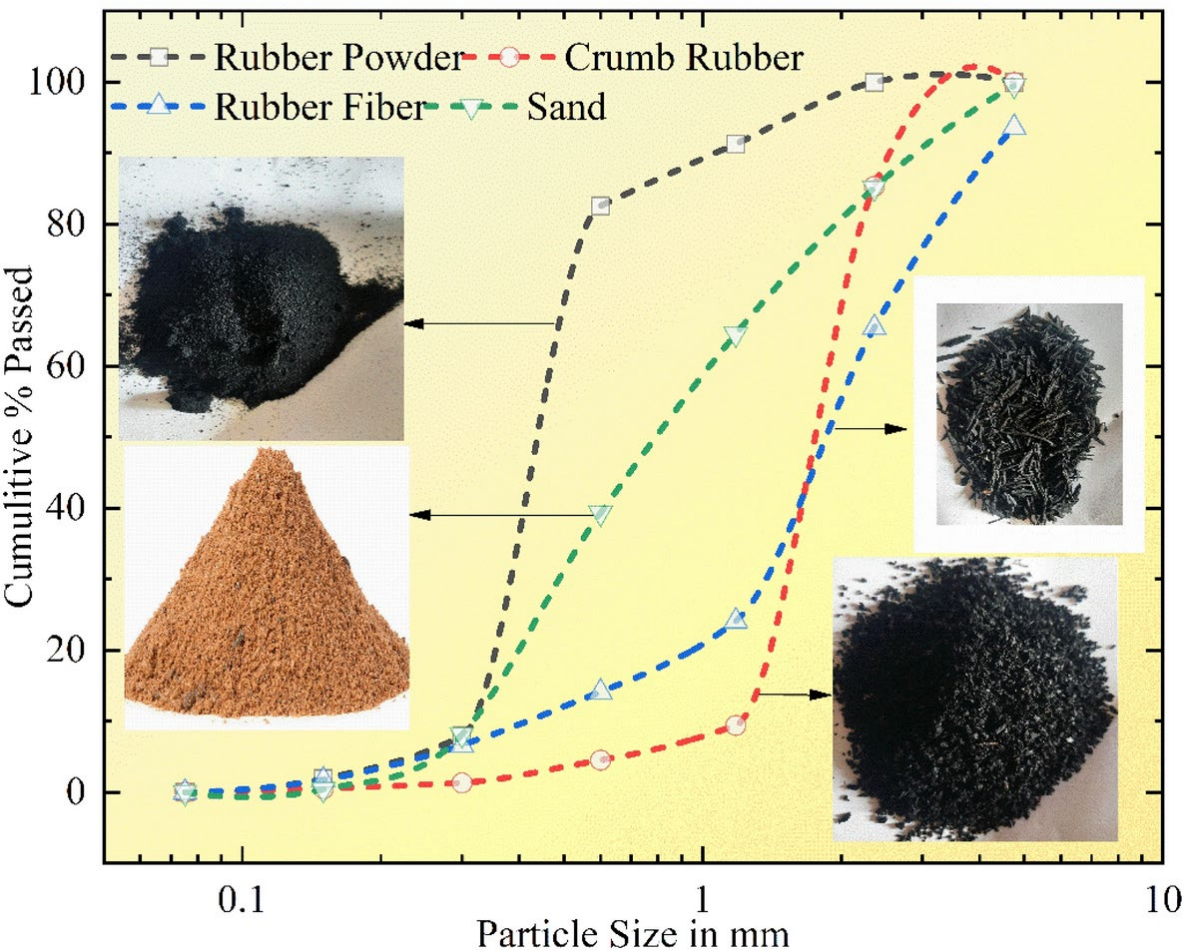
Sieve analysis of rubber particles and sand.

**Fig. 4:**
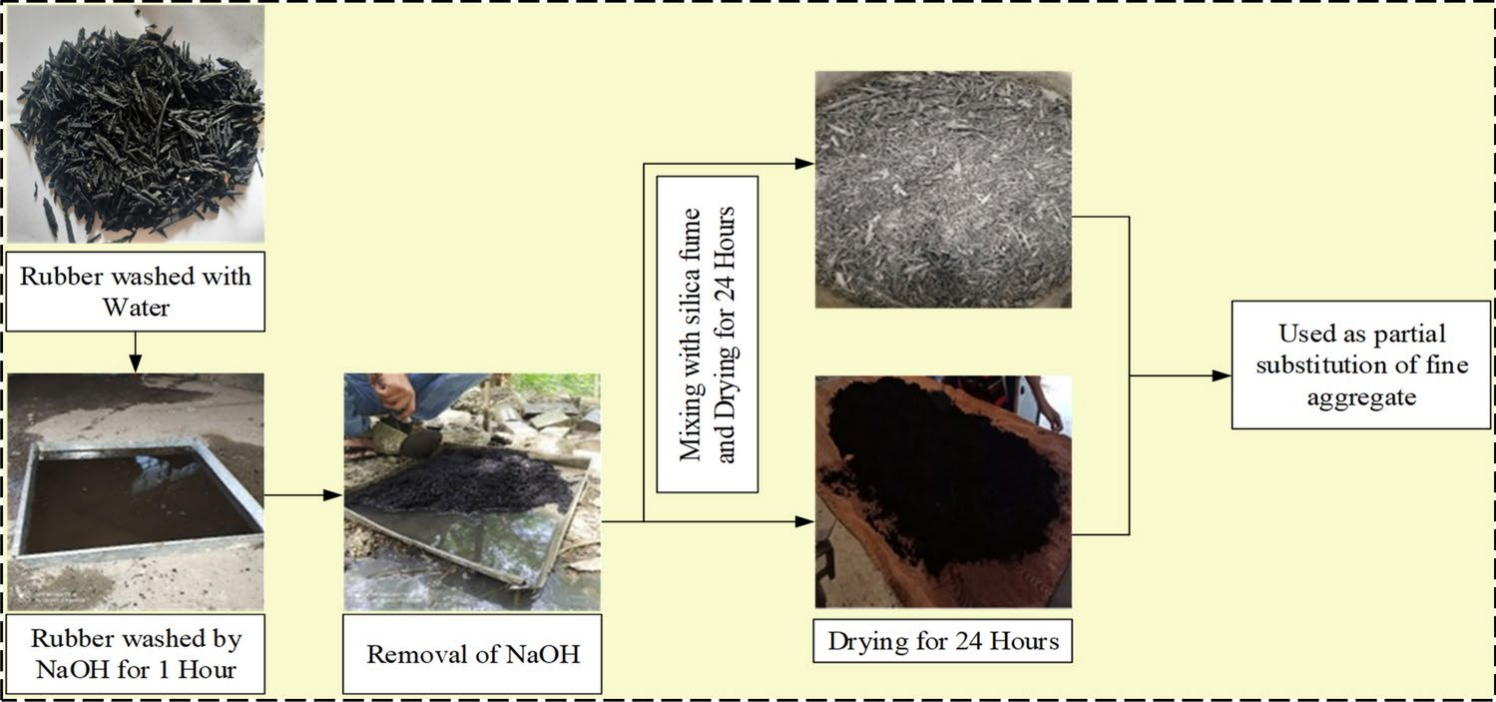
Pre-treatment processes of rubber particles by NaOH and by NaOH with silica fume.

**Fig. 5:**
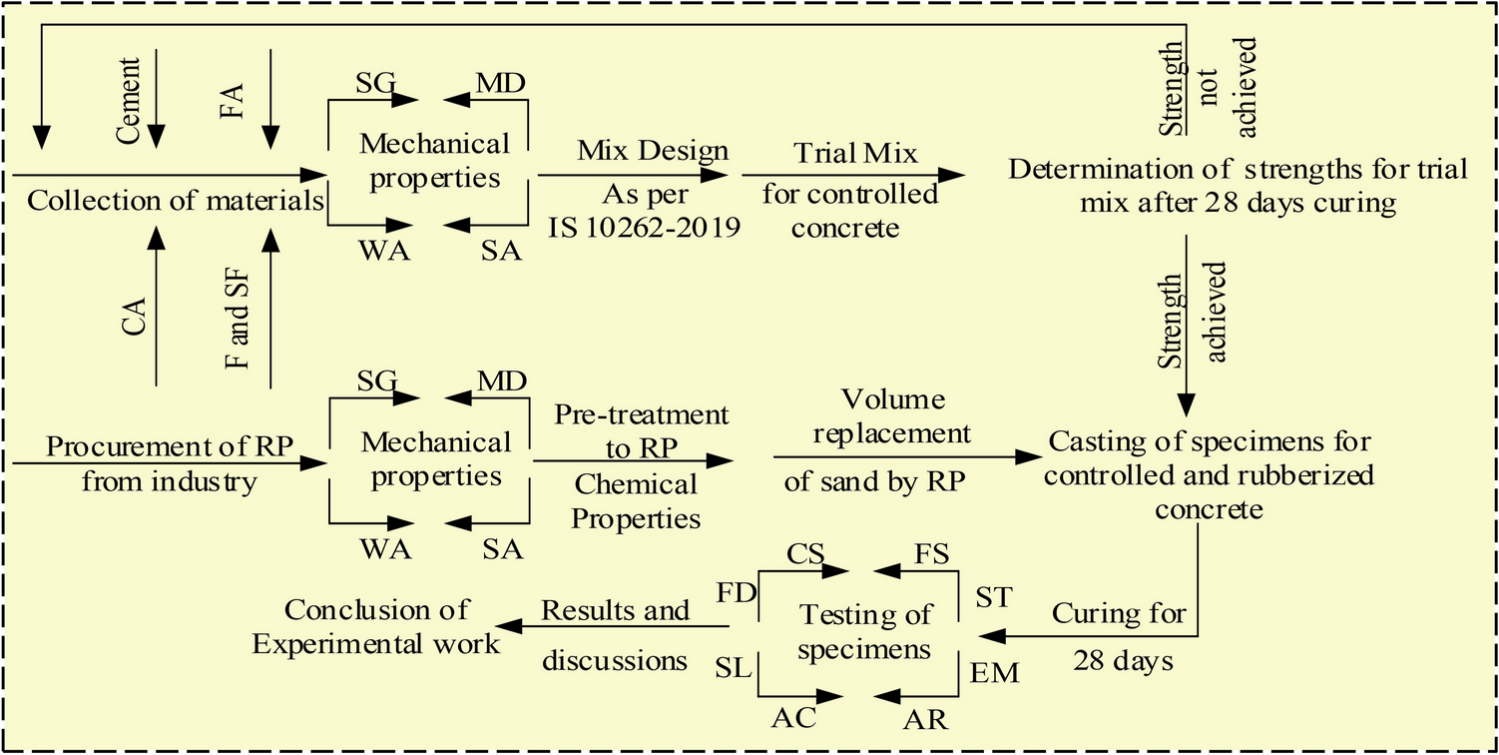
Methodology for experimental work Fine aggregates (FA), Coarse aggregates (CA), Fly ash (F), Silica fume (SF), Rubber Particles (RP), Specific Gravity (SG), Water Absorption (WA), Sieve Analysis (SA), Mass density (MD), Fresh density (FD), Slump Test (SL), Air content (AC), Compressive strength (CS), Flexural strength (FS), Split tensile strength (ST), Elastic Modulus (EM).

This experimental study on concrete was conducted using Ordinary Portland cement (OPC) of 3.15 g/cc specific gravity, referring to IS 269^40^. Fine aggregates with the grading of zone II and a specific gravity of 2.65 g/cc were utilized, and crushed angular coarse aggregates with a combination of 10 and 20 mm sizes are included by following Indian Standards^41–43^. To achieve the desired slump of concrete, super-plasticizer “Viscoflux 5507” having a specific gravity of 1.08 g/cc was used, and SF and fly ash were amalgamated as a cementitious powder to maintain maximum OPC cement content as per IS 456^44^. All necessary tests on aggregates were performed before their utilization in concrete. Figure 1 shows various tests performed on aggregates and rubber particles. Along with this for preparing RC, RP of size S-1, 300 microns, and specific gravity of 0.68 g/cc, CR of size S-2, from 300 microns to 1.18 mm, and specific gravity of 0.81 g/cc, and RF of size S-3, 1.18–2.36 mm with a length of fibers as 20 mm and specific gravity of 0.86 g/cc were procured from the nearby local rubber recycling industry, Fig. 2 depicts various forms of rubber particles utilized in this study as partial substitution of sand. Particle size distribution was carried out for sand and all forms of rubber particles and demonstrated in Fig. 3. The water absorption of rubber particles was found to be up to 0.5% and considering these subsequent dosages of superplasticizer were fixed in the mix design to achieve the desired workability. All the forms of waste tire rubber have been given treatment with NaOH of 1 Molarity (1 M) and included in concrete as a fractional switch of sand. The higher concentration of NaOH may deteriorate the rubber particles due to its etching action so 1 M solution is used in the study. The NaOH causes roughening of the surface of rubber partiles and also makes it hydrophilic from hydrophobic. Alongwith this the bonding property with cement matrix is also enhanced with removal of impurities present with rubber particles^17,18,45–47^. The silica fume is firstly introduced in the pretreatment technique by studying its cementitious properties and fineness which can enhance bonding properties. In addition, an arbitrary technique of mixing required SF with treated rubber before dry mixing of concrete elements to examine the influence on rubber particle-cement particle adhesion. The rubber elements were washed with potable water before initiating pre-treatment, then they were washed with NaOH and retained at ambient temperature for 24 h before casting. The treatment of rubber particles was demonstrated in Fig. 4. SF was also used to treat rubber particles along with NaOH. The SF added in the pretreatment process was 20% of the weight of rubber particles to be incorporated as a fractional switch of sand. The use of NaOH modifies the surface properties of rubber particles by breaking the carbon–sulfur bond. This breakdown emits carbon black and sulfides, the emitted carbon black from this process is almost inert, while the sulfides are generated in low quantity having minimal environmental impact. The methodology for experimental work in this research is illuminated in the flow chart displayed in Fig. 5. The chemical properties of rubber were determined and shown in Table 1. The Inductively Coupled Plasma Optical Emission Spectroscopy (ICP-OES) method is used to determine elements present in rubber particles like zinc, silicon, magnesium, and aluminum while, percentages of carbon, oxygen, and sulfur are derived using standard operating procedures of the laboratory.

**Table 1:** Chemical properties of rubber fibers.

Sr No	Test parameter	Test result (%)
1	Comprehensive element in rubber particles	
a	Zinc	1.17
b	Silicon	0.04
c	Magnesium	0.08
d	Aluminum	0.01
e	Carbon	78.07
f	Oxygen	4.42
g	Sulfur	1.89

### Mix proportioning of concrete

37 diverse groups of concrete mixes were prepared with variations in the particle sizes of waste tire rubber and with different treatment processes. Detailed mix proportions are mentioned in Table 2.

**Table 2:** Concrete mix proportions in kg/m^3^.

Sr. No	Mix	Cement	Fly ash	Silica fume	Water	Coarse aggregate	Sand	Rubber powder	Crumb rubber	Rubber fiber	Super-plasticizer	W\Cm ratio	Slump (mm)
1	CC0	450	122	37	155	1093	584	0	0	0	6.09	0.26	120
2	URP5	450	122	37	155	1093	555	7	0	0	6.21	0.26	120
3	URP10	450	122	37	155	1093	526	15	0	0	6.33	0.26	120
4	URP15	450	122	37	155	1093	497	22	0	0	6.45	0.26	120
5	URP20	450	122	37	155	1093	467	30	0	0	6.58	0.26	120
6	UCR5	450	122	37	155	1093	555	0	9	0	6.21	0.26	120
7	UCR10	450	122	37	155	1093	526	0	18	0	6.33	0.26	120
8	UCR15	450	122	37	155	1093	497	0	27	0	6.45	0.26	120
9	UCR20	450	122	37	155	1093	467	0	36	0	6.58	0.26	120
10	URF5	450	122	37	155	1093	555	0	0	9	6.21	0.26	120
11	URF10	450	122	37	155	1093	526	0	0	19	6.33	0.26	120
12	URF15	450	122	37	155	1093	497	0	0	28	6.45	0.26	120
13	URF20	450	122	37	155	1093	467	0	0	37	6.58	0.26	120
14	PRP5	450	122	37	155	1093	555	7	0	0	6.21	0.26	120
15	PRP10	450	122	37	155	1093	526	15	0	0	6.33	0.26	120
16	PRP15	450	122	37	155	1093	497	22	0	0	6.45	0.26	120
17	PRP20	450	122	37	155	1093	467	30	0	0	6.58	0.26	120
18	PCR5	450	122	37	155	1093	555	0	9	0	6.21	0.26	120
19	PCR10	450	122	37	155	1093	526	0	18	0	6.33	0.26	120
20	PCR15	450	122	37	155	1093	497	0	27	0	6.45	0.26	120
21	PCR20	450	122	37	155	1093	467	0	36	0	6.58	0.26	120
22	PRF5	450	122	37	155	1093	555	0	0	9	6.21	0.26	120
23	PRF10	450	122	37	155	1093	526	0	0	19	6.33	0.26	120
24	PRF15	450	122	37	155	1093	497	0	0	28	6.45	0.26	120
25	PRF20	450	122	37	155	1093	467	0	0	37	6.58	0.26	120
26	SPRP5	450	122	37	155	1093	555	7	0	0	6.21	0.26	120
27	SPRP10	450	122	37	155	1093	526	15	0	0	6.33	0.26	120
28	SPRP15	450	122	37	155	1093	497	22	0	0	6.45	0.26	120
29	SPRP20	450	122	37	155	1093	467	30	0	0	6.58	0.26	120
30	SPCR5	450	122	37	155	1093	555	0	9	0	6.21	0.26	120
31	SPCR10	450	122	37	155	1093	526	0	18	0	6.33	0.26	120
32	SPCR15	450	122	37	155	1093	497	0	27	0	6.45	0.26	120
33	SPCR20	450	122	37	155	1093	467	0	36	0	6.58	0.26	120
34	SPRF5	450	122	37	155	1093	555	0	0	9	6.21	0.26	120
35	SPRF10	450	122	37	155	1093	526	0	0	19	6.33	0.26	120
36	SPRF15	450	122	37	155	1093	497	0	0	28	6.45	0.26	120
37	SPRF20	450	122	37	155	1093	467	0	0	37	6.58	0.26	120

### Tests performed on concrete

#### Properties of fresh concrete

The workability of each set of concrete was determined and the hardened properties were assessed as per standards^48^ for 28 and 90 days. 28 and 90-day strength results were shown in this study. The appropriate conclusions were then obtained after comparing the strengths of the control mix and RC mixes containing untreated rubber particles with the strengths of concrete containing various rubber sizes.

ASTM C138^37^ was referred to compute the densities of the control concrete mix and the RC. The density of fresh concrete was calculated and compared with the theoretical density by determining yield. From the density, the air content of concrete was evaluated.

#### Compressive strength

The compressive strength was computed for cubes of size 150 mm and on 150 mm diameter cylinders with a length of 300 mm conforming to IS 516 and IS1199^34,49^. Compressive strength was intended for cylindrical specimens; an extensometer fitted with a calibrated dial gauge was connected to concrete cylinders as shown in Fig. 7c to measure the displacement after the application of loading on specimens. Static elastic modulus was determined from the details retrieved from cylindrical compressive strength.

#### Flexural and split tensile strength

The flexural strength and indirect tensile strength were determined in consideration of norms specified in IS 516 and IS 5816^34,35^. 100 mm × 100 mm × 500 mm rectangular beams were employed to take out the flexural strength. Splitting tensile strength was performed on 150 mm diameter cylinders with a length of 300 mm. The flexural and split tensile strength of each mix was calculated using the average of three specimens.

#### Abrasion resistance

Abrasion is the behavior of a construction element when it is subjected to destructive forces such as scraping, rubbing, etc. of an object on a surface, which results in the surface wearing. Concrete tiles with a length and width of 70.6 mm and 25 mm thickness were cast and tested on the Dorry abrasion testing machine. The abrasion resistance of concrete tiles was computed by following the clauses of IS 15658^39^ and IS 1237^36^. Loss in volume is measured as per IS 15658^39^ and the depth of wear is measured after the completion of revolution cycles by considering the loss of mass by IS 1237^36^. In this study both methods were studied and resistance to abrasion was described as the depth of wear for concrete.

#### Dynamic modulus of elasticity

The dynamic elastic modulus was measured using the ultrasonic pulse velocity device following American Standards^38^. The longitudinal distance between two transducers applied to the two different faces of the testing cube specimen and the transit time required for the longitudinal wave was noted, which in turn was used as basic required data to calculate the velocity of the wave. The further calculation procedure for assessing the elastic modulus of the control and altered concrete process explained by researchers^46,50^ has been opted.

#### Water absorption of concrete

The water absorption of concrete was assessed by following BS1881^51^. The oven-dried concrete specimens of diameter and length of 75 mm were used. After removing specimens from the oven, they are allowed to cool for 24 ± 0.5 h. The mass for each specimen was noted instantly after cooling and then the specimens were dipped utterly in water for 30 ± 0.5 min. After this drying of specimens with the help of cloths to remove free water from the surface and then the weighing of each specimen was carried out. The correction factor was assessed using the following equation.

$$Correction Factor (C.F.)= \frac{Volume}{Surface \,Area \times 12.5}$$

The correction factor is necessary to calculate when the specimen has a size different than the 75 mm diameter and varying height. As the specimens used in this study is 75 mm in diameter with 75 mm in height, the correction factor used was 1.

## Results and discussions

**Fig. 6:**
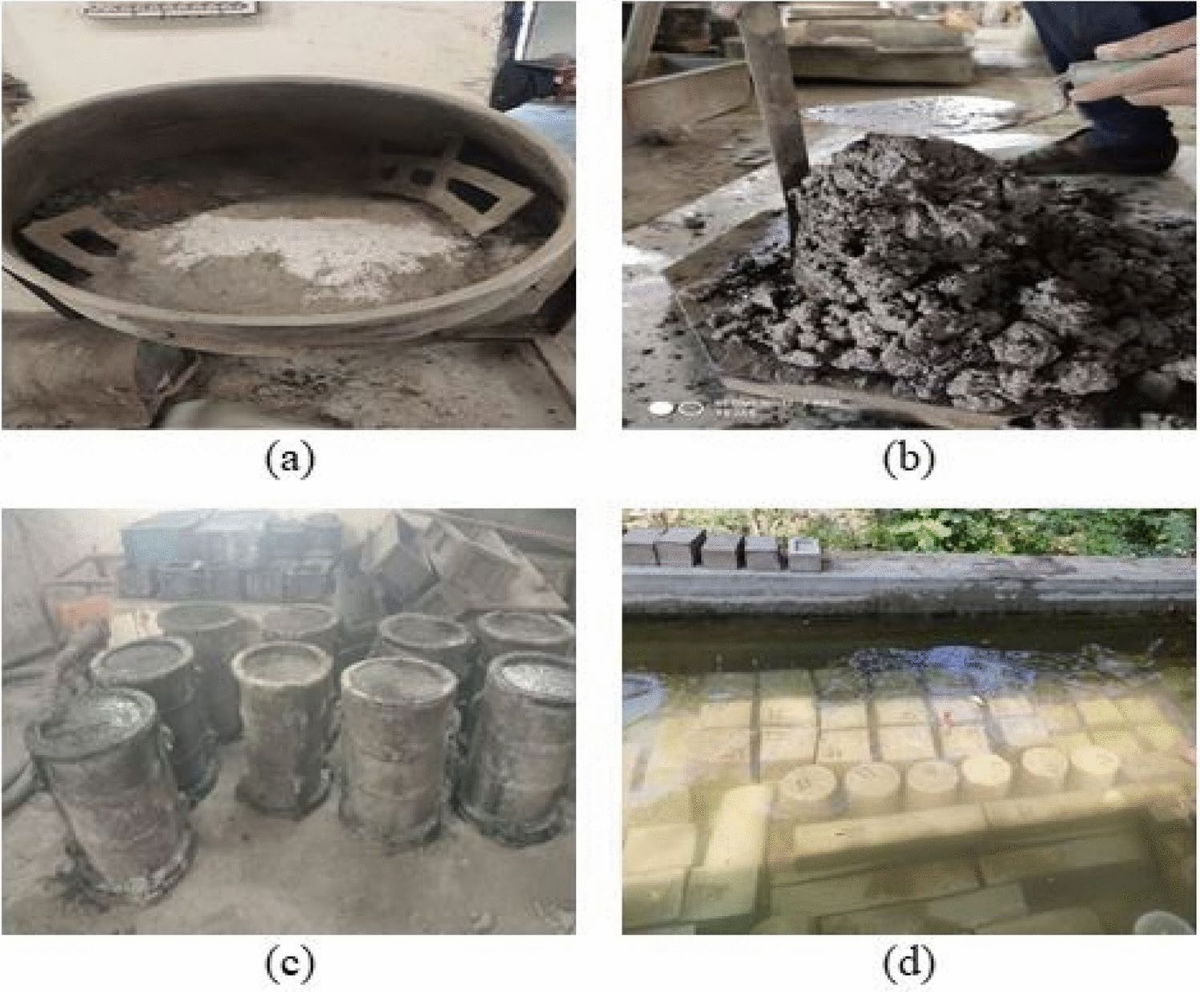
Casting of Specimens (**a**) Dry Mixing, (**b**) Slump Determination, (**c**) Casting, and (**d**) Curing.

**Fig. 7:**
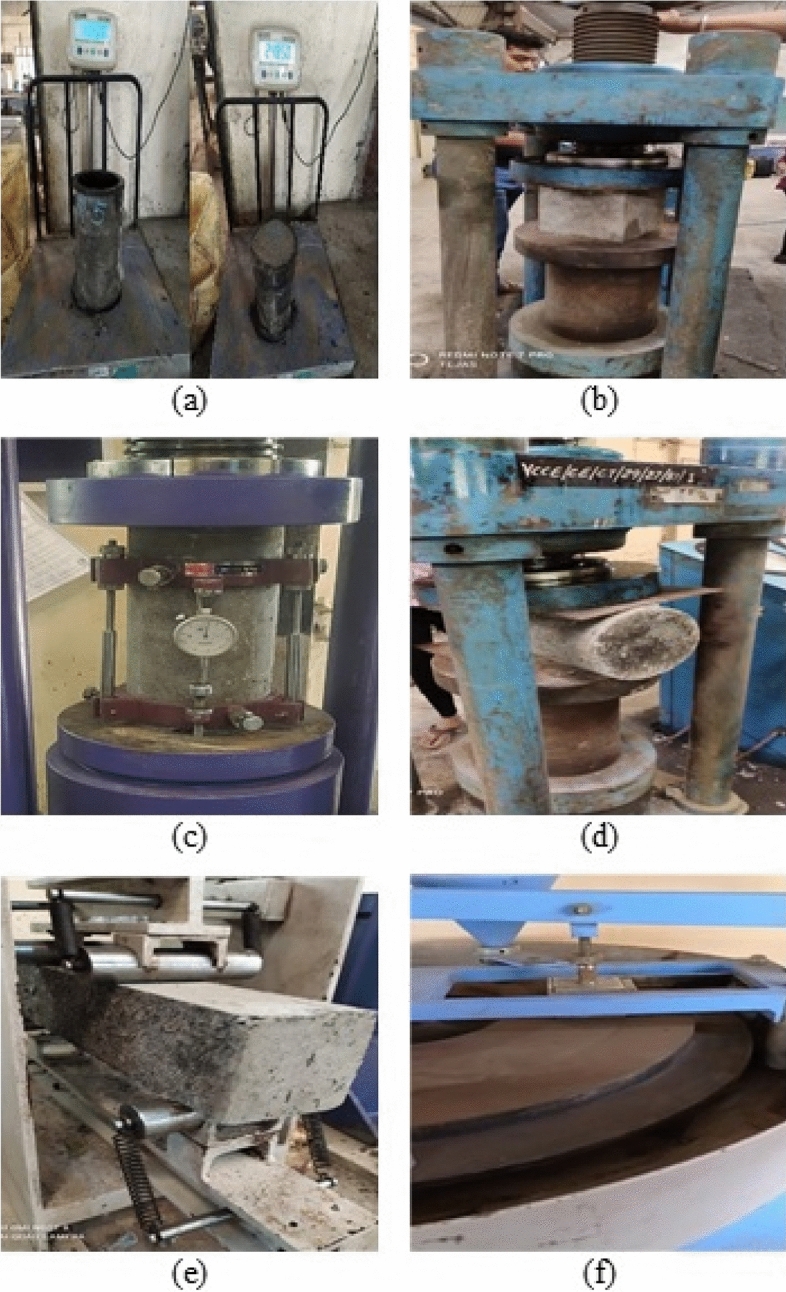
Testing of Specimens (**a**) Fresh Density of Concrete, (**b**) Compressive Strength, (**c**) Cylindrical Compressive Strength, (**d**) Splitting Tensile Strength, (**e**) Flexural Strength, (**f**) Abrasion Resistance Test, (**g**) Dynamic Modulus of Elasticity using UPV.

According to IS 516^34^, the different tests were conducted at 28 and 90 days. Some tests were conducted on freshly prepared concrete and all test results are described below. All concrete mixes’ slump values were maintained at 120 mm by varying the super-plasticizer dosages as indicated in Table 2. The casting and testing of different specimens and determination of workability are depicted in Figs. 6 and 7 represents the testing of various specimens in this experiment.

### Properties of fresh concrete

**Fig. 8:**
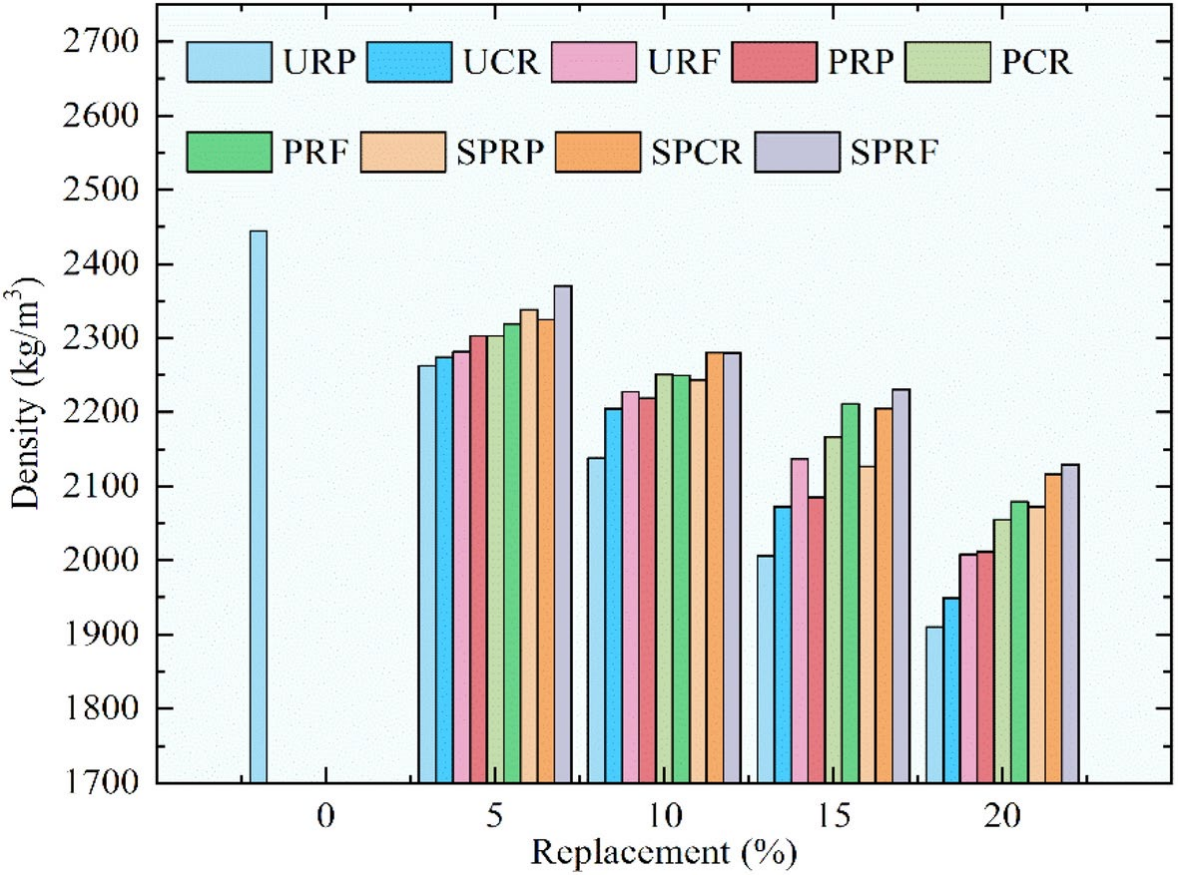
Comparison of density of fresh concrete.

**Fig. 9:**
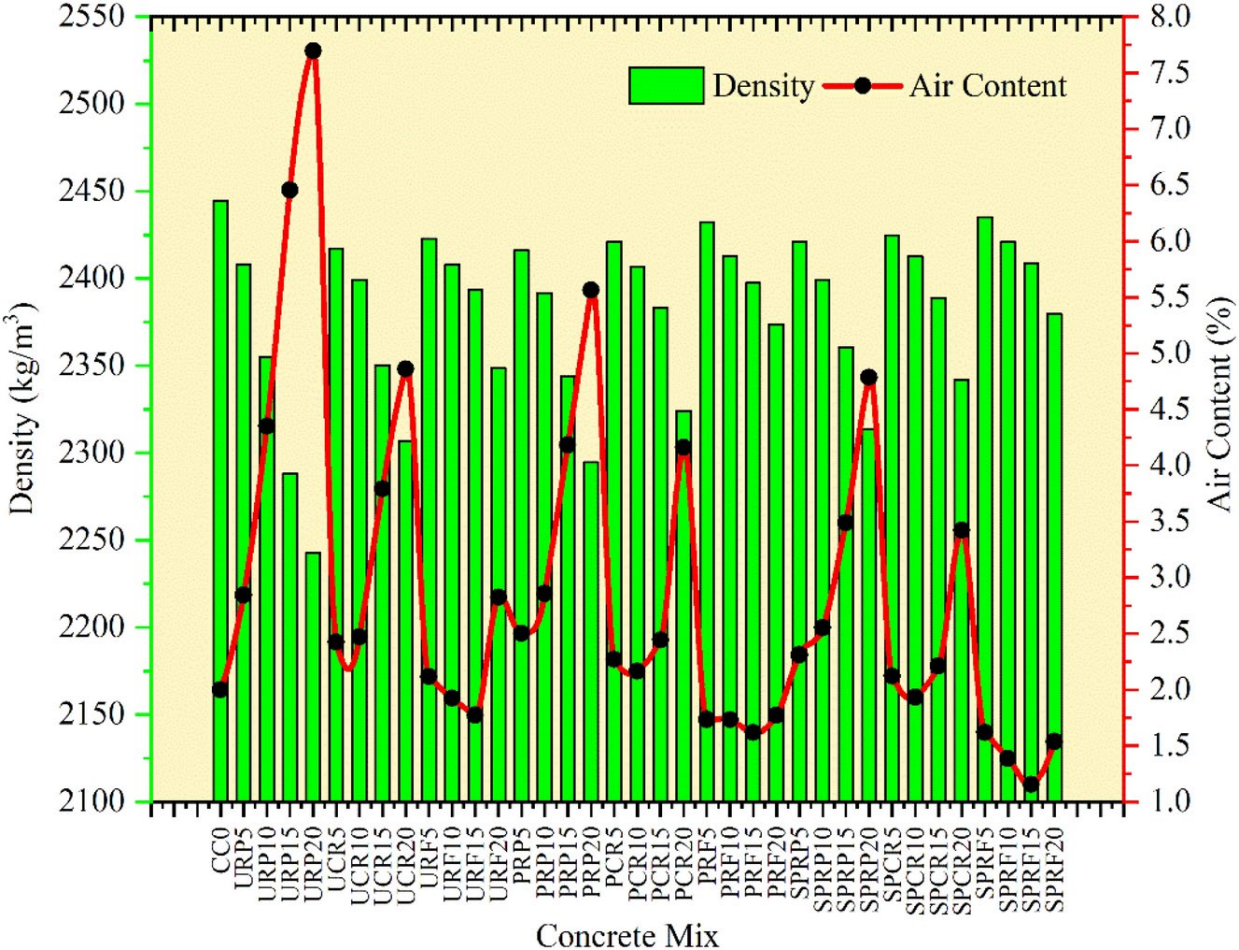
Density and air content for concrete.

To determine the air content and fresh concrete density according to ASTM C138^37^, the concrete mixtures were analyzed. The measuring vessel of known volume (*V*_*m*_) was taken and its mass was noted as (*M*_*c*_) then it was filled up to the top surface in equal layers. Every layer was compressed using a needle vibrator, the measuring vessel was cleaned from the outer face and the top was made plane. Then the mass of the measuring vessel along with the concrete was documented as (*M*_*m*_). By subtracting the mass *M*_*c*_ from *M*_*m*_ and dividing it by (*V*_*m*_) density (*D*) was calculated for all concrete mixes. Theoretical density (*T)* was determined and subsequently, the air content was calculated. The records of density and air content are shown in Figs. 8 and 9.

The reduction in densities of concrete mixes incorporating different forms of rubber as limited substitution of sand was observed. The obtained results were similar to the expected results as the densities of S-1, S-2, and S-3 are lower than that of the density of sand. The partial substitution of rubber particles with pre-treatment by NaOH and by NaOH plus silica fume has shown a lesser reduction in densities as linked to the concrete with untreated rubber particles for all varying sizes of rubber. More fine particles of rubber without any treatment reduced the density highly. The reduction in densities of concrete was 8.2%, 5.6%, and 3.9% for the 20% replacement of sand by URP, URC, and URF respectively. Similarly, the lessening of densities was 6.1%, 4.9%, and 2.9% for the 20% substitution of sand by PRP, PRC, and PRF which was pre-treated by NaOH. Rubber particles with arbitrary treatment invented in this research by using NaOH and silica fume had shown the most promising results as a decrease in densities was the lowest at 5.3%, 4.2%, and 2.6% correspondingly for SPRP, SPRC, and SPRF. In the research experiment^28^ the reduction in density was around 16.5% for the 20% replacement of sand in concrete with silica fume, while in the present study, the reduction in densities was noted below 6% due to the pre-treatment of rubber particles. Similarly, the observations recorded in another study^19^ for a reduction in density are around 3.5% for 20% replacement of sand and in this study, the density was reduced by 2.6% for 20% replacement of sand by SPRF.

The air content for concrete mixes was evaluated by opting for the procedure stated in^37^, and the results obtained were explained in Fig. 9. The air content of concrete mixes was plotted against the densities obtained for the control mix and RC. Every type of concrete mix containing untreated rubber particles had its air content altered at random, and it was discovered that this air concentration was highest when compared to the other concrete mixes and the control mix. Pre-treatment process caused the reduction in air content. The maximum air content was found to be 7.7% for 20% replacement of sand by URP.

### Compressive strength

The compressive strength for concrete mixes was computed according to the Indian code^34^. The concrete cubes used in this research were of size 150 mm. The placing of concrete to prepare specimens was done as per procedure, concrete was placed in three layers and compacted by a surface vibrator. Curing for all cubes was done for 28 and 90 days. Exterior water and shingle were rubbed off from specimens and any prominent fins were removed. The weight of all concrete cubes was noted and then they were checked using a compression testing machine. When the cube failed after applying compressive load at the rate of 140 kg/sq cm/minute, the final reading of load was noted and it was divided by its area. The average compressive strength for three specimens of each proportion was taken as compressive strength for that concrete mix.

**Fig. 10:**
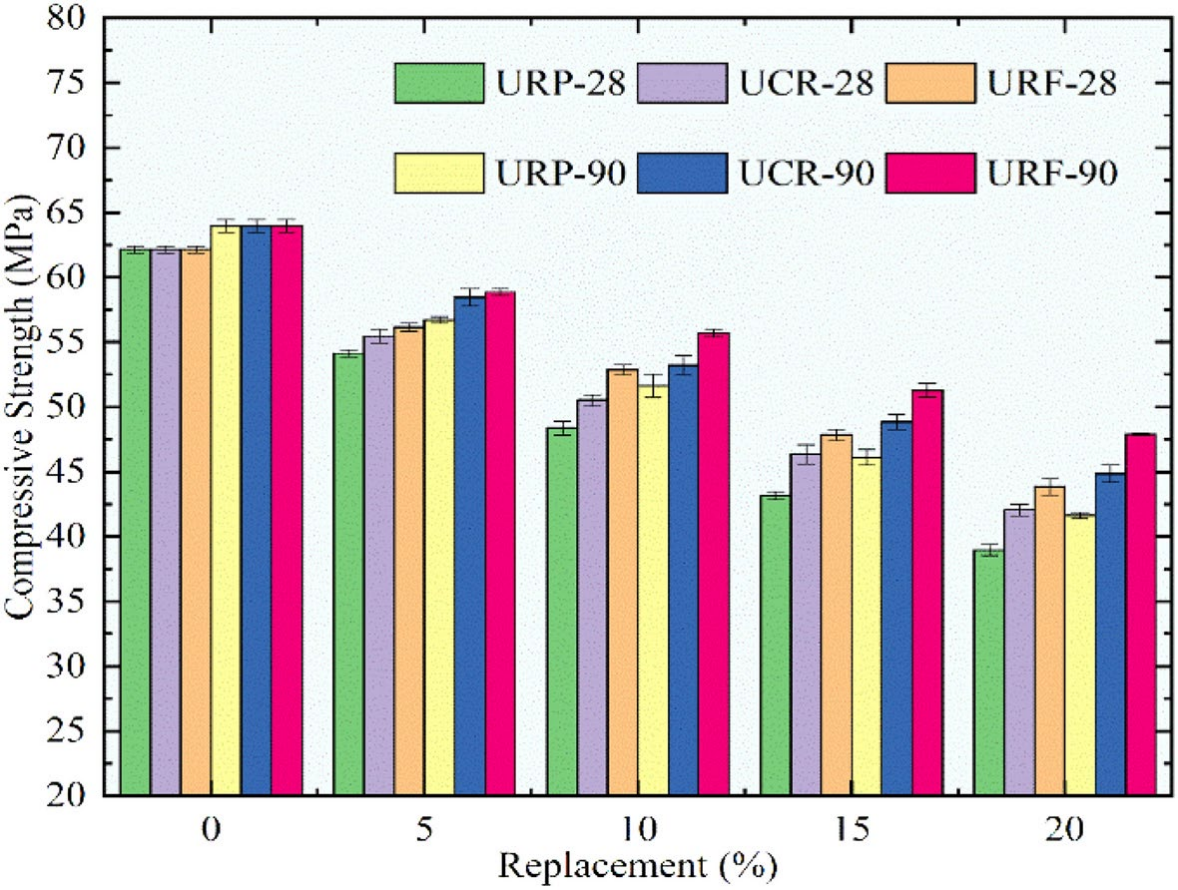
Variation in compressive strength for control concrete and RC mixes with untreated rubber particle.

Figure 10 demonstrated that the compressive strength declined linearly with the raise in rubber particles. The mean compressive strength for the control concrete was found to be 62.1 MPa and 63.9 MPa subsequently for 28 and 90 days. The optimum lessening in strength was found to be for the URP20 mix with a reduction of 37.3% and 34.9% for 28 and 90 days. At 20% replacement RC mixes with untreated CR and untreated RF showed a decline of 32.3% and 29.4% at 28 days while a slight improvement was recorded for compressive strength at 90 days in comparison with 28 days. The reduction for UCR20 and URF20 at 90 days was 29.9% and 25.1%. The reduction in compressive strength could be because of the weak interfacial binding between cement paste and untreated rubber particles as the rubber particles have smooth surfaces and they behave as hydrophobic particles.

**Fig. 11:**
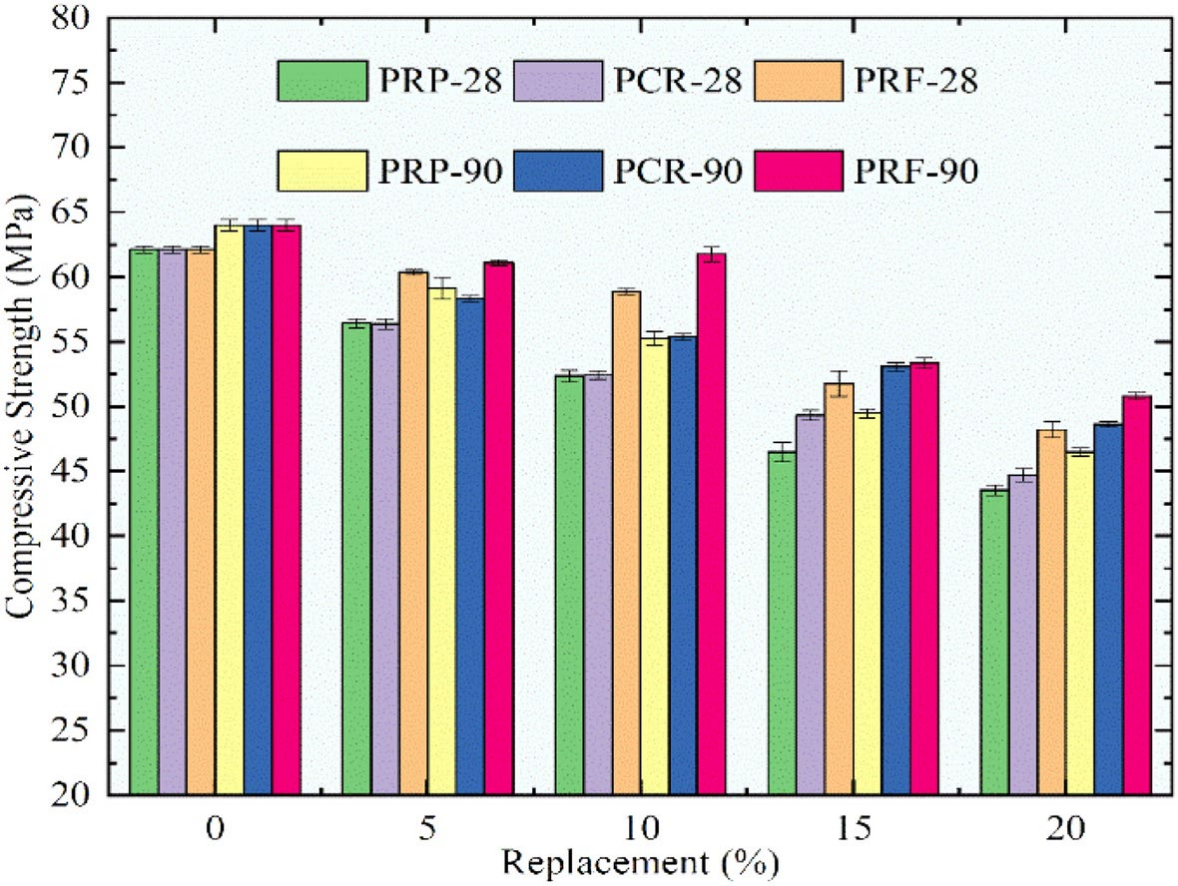
Variation in compressive strength for control concrete and RC mixes with NaOH pretreated rubber particles.

The deviation in compressive strength for the control mix and RC mixes having rubber particles pretreated with NaOH after curing for 28 and 90 days is depicted in Fig. 11. The same tendency of lessening in compressive strength was detected for the upsurge in rubber content. The decrement ratio was lessened significantly when linked to the strength of RC mixes with untreated rubber particles. The highest loss was noted for the mix PRP20 as the compressive strength reduced by 29.9% and by 27.3% respectively for 28 and 90 days when associated with the control concrete. The RC mixes PCR20 and PRF20 exhibited a decline in strength at 28%, 24%, 22.4%, and 20.6% consequently for 28 and 90 days. The RC mix PRF5 exhibited a compressive strength of 60.3MPa at 28 days and 61.1 MPa for 90 days, while the mix PRF 10 showed a strength of 61.7 MPa, which was more than the characteristic compressive strength assumed in this part of the study. From Fig. 11 it was seen that the strength decreased more for RP than CR and RF. The RC mixes with RF showed promising strengths in comparison to the RC mixes with RP and CR.

**Fig. 12:**
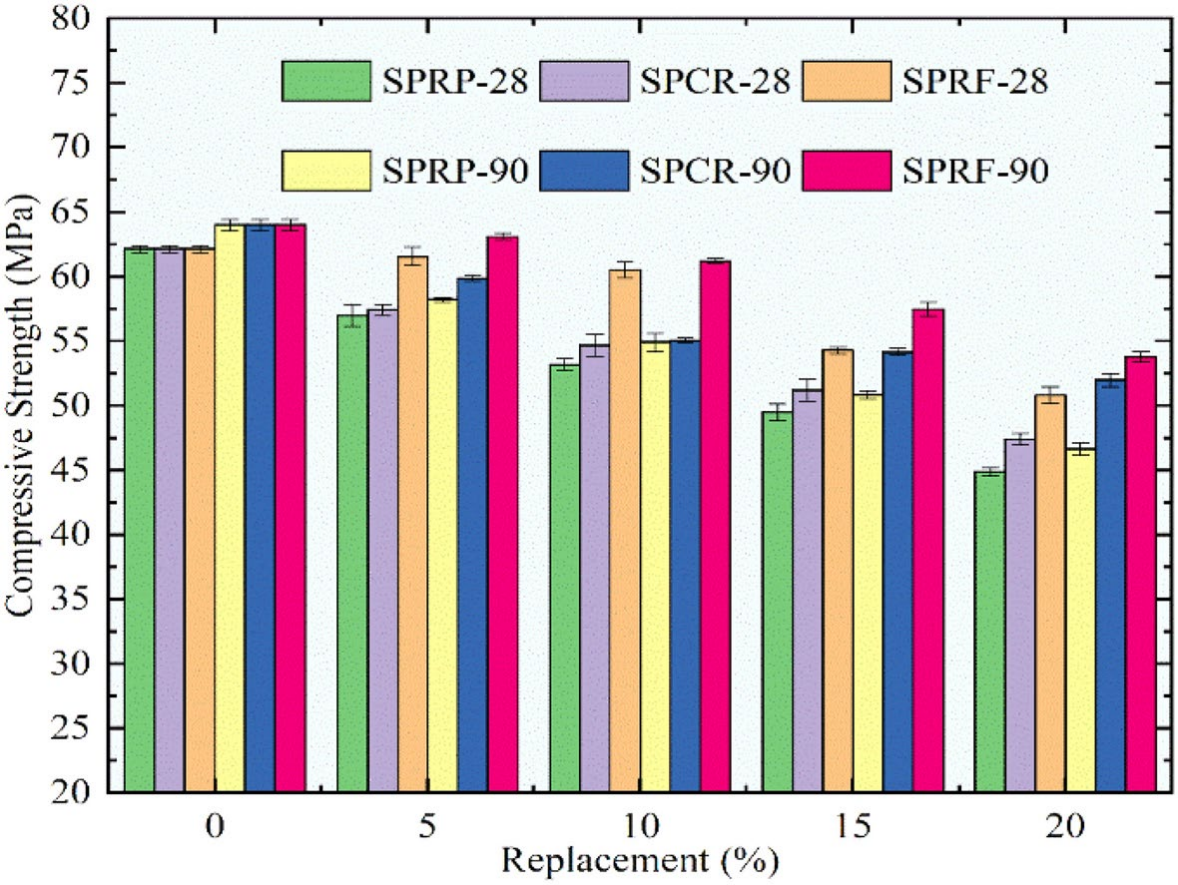
Variation in compressive strength for control concrete and RC mixes with NaOH + silica fume pretreated rubber particle.

Figure 12 shows the deviation in compressive strength for the control mix and the RC mixes having rubber particles pretreated with immersion in NaOH and using silica fume. The RC mixes showed better performance up to 10% substitution of sand using rubber particles pretreated by this technique. A declining trend was noted for finer rubber particles, while the RC mixes with pretreated CR and RF showed good compressive strength. The maximum decrease of 27.7% and 27.1% was recorded for the mix SPRP20 at 28 and 90 days respectively in comparison to the compressive strength of the control mix. For the SPCR20 mix a decrease of 23.7% and 18.7% was noted. The extreme dropping off for the SPRF20 mix was observed as 18.2% and 15.9% at 28 days and 90 days. The RC mixes SPRF5 and SPRF10 showed a compressive strength of 61.5 MPa and 60.5 MPa at 28 days and 63.0 MPa and 61.2 MPa at 90 days of curing, which is higher than the characteristic compressive strength.

From the results obtained for compressive strength for all RC mixes it was seen that the pretreated rubber particles performed better than untreated rubber particles. promising results were obtained for RF than CR and RP. Hence it can be stated that the rubber particles in the form of fibers and pretreated with NaOH, and silica fume have the potential to give better strength up to 10% substitution of sand.

**Fig. 13:**
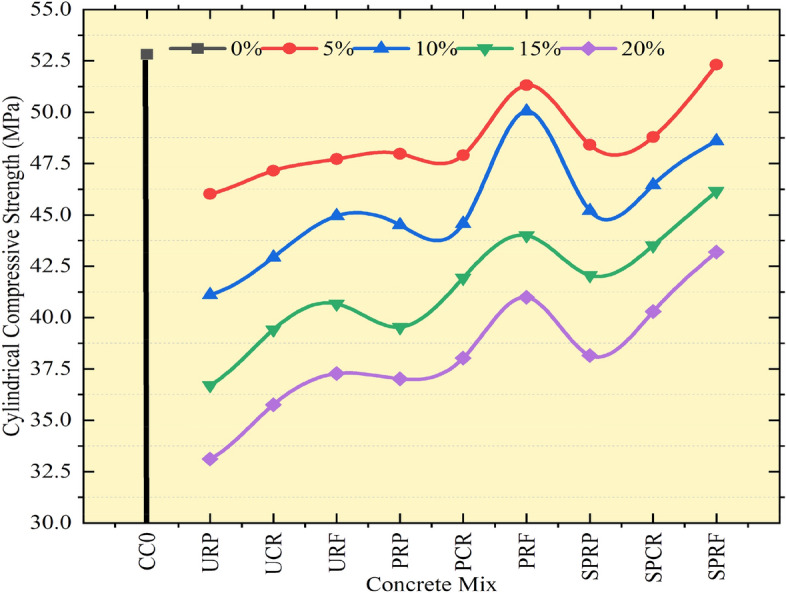
Variation in cylindrical compressive strength for concrete mixes.

Compressive strength on cylindrical specimens was carried out according to standards^34^ with the use of an extensometer fitted with a dial gauge to determine the displacement in the cylinder after the application of compressive load by a compression testing machine as shown in Fig. 13. Concrete cylinders for all mixes cured for 28 days was tested and strengths were calculated for each mix as an average of three specimens. The load–displacement outcomes were noted and composed for concrete mixes with SPRF from 0 to 20% replacement of sand, for different load values as described in Table 3. The displacements of concrete mixes containing rubber particles were increased enormously in parallel to the normal concrete for SPRF20 the deflection in the concrete cylinder was increased approximately by 453.8% at the same loading of 510 kN when linked to the control concrete. From the outcomes, it was noticed that RC can enhance the strain rate for concrete. In comparison to the control concrete, the compressive strength of every cylindrical specimen dropped. The decrement in cylindrical compressive strength of around 20.6% was observed for the concrete mix SPRF20 in contrast to the control mix with no rubber particles. Authors^9^ experimented on RC and found that due to increased strain rate the energy absorption of concrete is enhanced at higher rubber replacement ratios.

**Table 3:** Load–displacement relation for concrete mixes with SPRF and control concrete.

Load (KN)	% Replacement of sand by rubber fiber pretreated with NaOH and silica fume				
	0%	5%	10%	15%	20%
30	0.02	0.02	0.02	0.03	0.03
60	0.04	0.03	0.04	0.07	0.08
90	0.04	0.06	0.06	0.12	0.13
120	0.05	0.08	0.07	0.15	0.23
150	0.06	0.09	0.09	0.17	0.27
180	0.07	0.09	0.10	0.19	0.32
210	0.07	0.10	0.12	0.23	0.35
240	0.08	0.12	0.13	0.27	0.42
270	0.08	0.13	0.16	0.30	0.43
300	0.09	0.14	0.18	0.35	0.45
330	0.10	0.16	0.20	0.40	0.49
360	0.10	0.16	0.21	0.42	0.54
390	0.11	0.18	0.23	0.47	0.59
420	0.12	0.20	0.25	0.50	0.60
450	0.13	0.21	0.27	0.52	0.65
480	0.13	0.22	0.28	0.56	0.71
510	0.13	0.23	0.30	0.59	0.72
540	0.14	0.24	0.32	0.62	0.77
570	0.15	0.25	0.37	0.66	0.80
600	0.16	0.27	0.39	0.69	0.84
630	0.17	0.28	0.41	0.71	0.86
660	0.18	0.28	0.43	0.73	0.87
690	0.18	0.30	0.46	0.74	0.89
720	0.19	0.30	0.47	0.76	0.90
750	0.19	0.32	0.49	0.79	0.90
780	0.20	0.33	0.52	0.80	0.00
810	0.21	0.36	0.54	0.80	0.00
840	0.21	0.37	0.58	0.00	0.00
870	0.22	0.39	0.61	0.00	0.00
900	0.23	0.40	0.61	0.00	0.00
930	0.23	0.00	0.00	0.00	0.00

### Flexural strength and split tensile strength

**Fig. 14:**
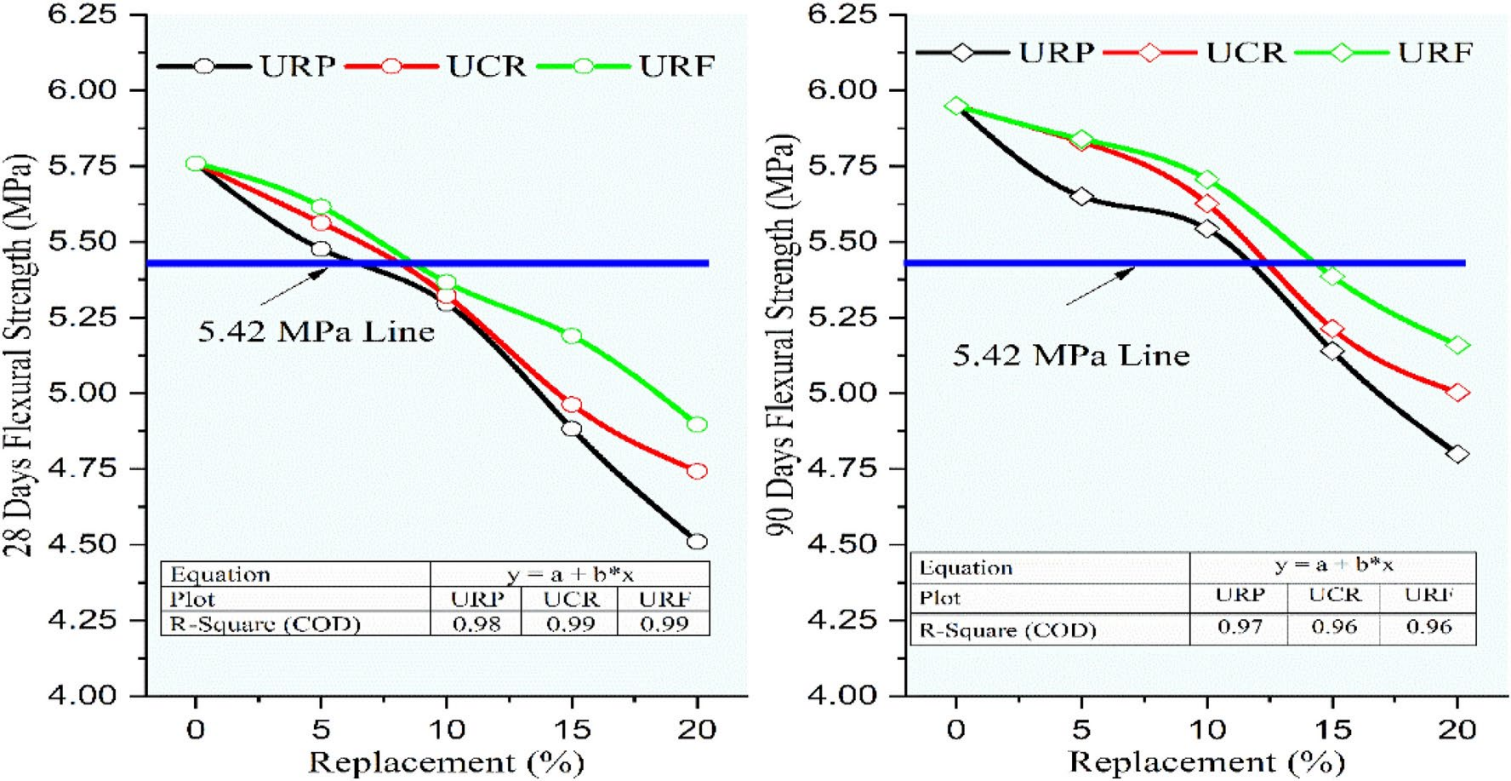
Variation in flexural strength for control concrete and RC mixes with untreated rubber particle.

The flexural and split tensile strengths were determined to conform to Indian standards^34,35^. For determining the flexural strength rectangular beams were assessed subsequently after 28 and 90 days of curing, the beam was supported by two identical roller shafts spaced at 400 mm distance, and the loading was applied through another two roller shafts touched at the top of the beam. The modulus of rupture was determined by noting the crack formation as per criteria, the average of three specimens was considered to finalize the flexural strength. From Fig. 14, the flexural strength for RC mixes was found to be decreased linearly with an increment in the rubber content. For 28 and 90 days the flexural strength showed better performance for RC mixes with RF than the RC mixes with CR and RP. The RC mixes URP5, UCR5, and URF5 at 28 and 90 days, while URP10, UCR10, and URF10 at 90 days exhibited strength more than the minimum required flexural strength i.e. 0.7 times the square root of characteristic compressive strength, which is equal to 5.42 MPa for M60 grade concrete. Among the RC mixes maximum strength was obtained for the mix URF5 as 5.62 MPa and 5.84 MPa while the control concrete showed a strength of 5.76 MPa and 5.95 MPa at 28 and 90 days respectively. The mix URF10 showed a minimal decrease of 6.83% and 4.09% than the control concrete. The maximum decrease was seen for 20% replacement as URP20, UCR20, and URF20 showed a strength of 4.5 MPa, 4.8 MPa, 4.7 MPa, 5 MPa, 4.9 MPa, and 5.16 MPa at 28 and 90 days respectively with the corresponding decrease of 21.70%, 19.33%, 17.65%, 15.91%, 14.99%, and 13.28% at 28 and 90 days.

**Fig. 15:**
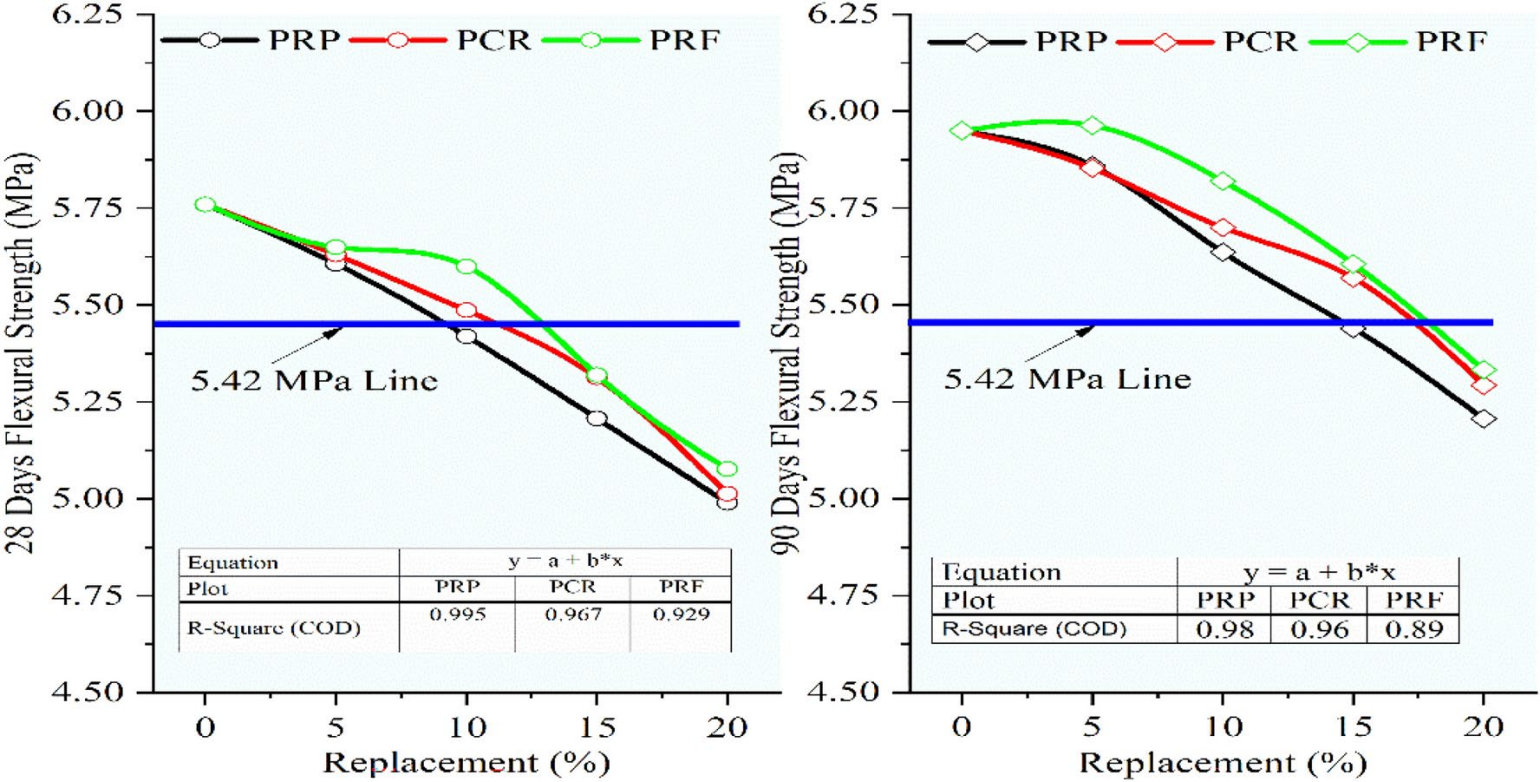
Variation in flexural strength for control concrete and RC mixes with NaOH pretreated rubber particles.

Figure 15 demonstrated the decrement in the flexural strength with an increment in the rubber content for RC mixes having rubber particles pretreated with NaOH. The flexural strength of RC mixes PRP5, PCR5, and PRF5 showed almost the same strength at 28 days with a marginal increase with the increase in sizes of rubber particles. The mixes PRP5, PCR5, PRF5, PCR10, and PRF10, showed strength higher than 5.42 MPa at 28 days. While the mixes with 5 and 10% replacement for all sizes and mixes with 15% substitution containing CR and RF particles showed a strength of more than 5.42 MPa at 90 days. The maximum decline in strengths was noted for the RC mixes with 20% replacement as the strengths were reduced by 13.37%, 12.49%, 12.96%, 11.04%, 11.86%, and 10.36% for RP, CR, and RF particles at 28 and 90 days respectively. The reduction in strength was reduced as compared to the strengths of RC mixes having untreated rubber particles.

**Fig. 16:**
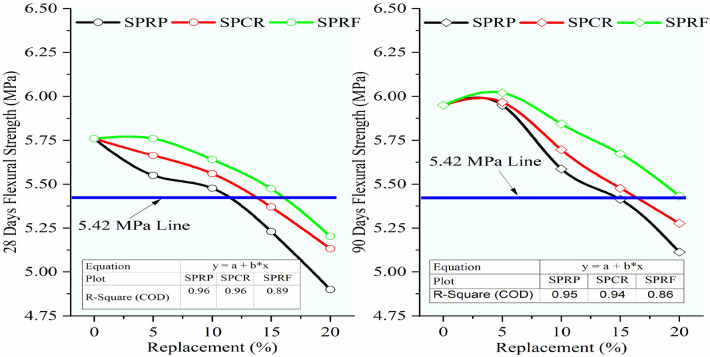
Variation in flexural strength for control concrete and RC mixes with NaOH + Silica fume pretreated rubber particle.

Referring to Fig. 16, it was seen that the same trend of decrease in flexural strength was continued for the RC mixes having rubber particles pretreated with the new pretreatment technique adopted in the study using NaOH + silica fume. But the decrement ratio was curtailed up to 55% when compared to the lessening in the strength of RC mixes having untreated rubber particles. The optimum loss was noted for RC with 20% SPRF was found to be 9.66% and 8.68% while for untreated RC mixes it was 21.70% and 19.33% respectively. The RC mixes SPRP5, SPCR5, and SPRF5 gained flexural strength higher than the control mix at 90 days. Up to 10% switch of sand using rubber particles pretreated with this method the optimal decline of 6.11% was noted for the mix SPRP10 at 90 days, while the mix SPRF10 showed a maximum decrease of only 2.08% at 28 days and 1.79% at 90 days.

**Fig. 17:**
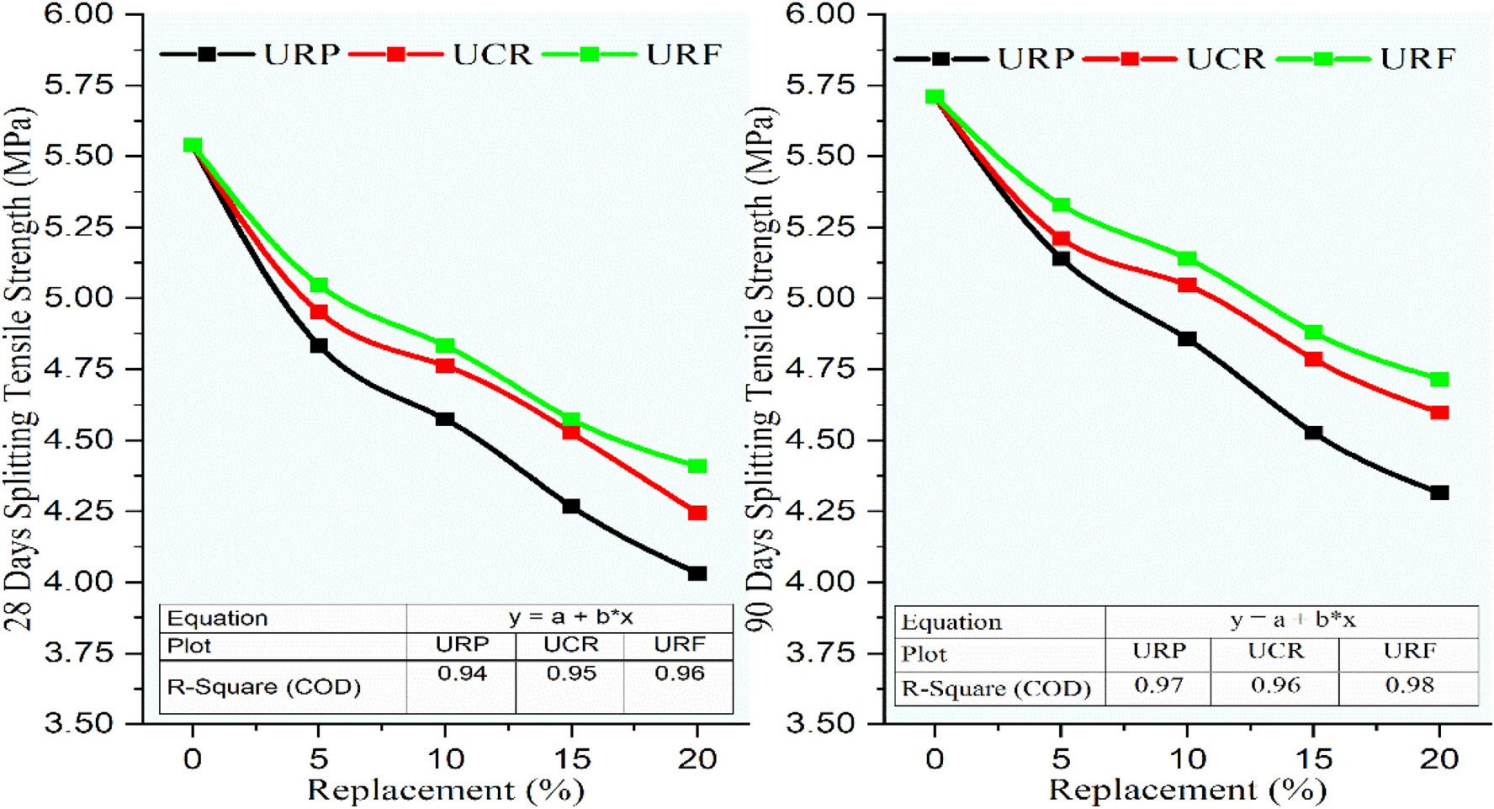
Variation in splitting tensile strength for control concrete and RC mixes with untreated rubber particle.

The splitting tensile strength for RC mixes having untreated rubber particles was realized to be decreased with rise in the rubber content. Referring to Fig. 17, the splitting tensile strength for the control concrete was gained to be 5.54 MPa and 5.71 MPa subsequently for 28 and 90 days. The determined lessening was observed for 20% substitution of sand using rubber particles for URP20 the decrement was 27.23% and 24.38% while for UCR20 and URF20 the strength was decreased by 23.40%, 19.42%, 20.43%, and 17.36% at 28 and 90 days when equated to the control concrete. From these results, it was seen that the reduction ratio decreased with an increase in the size of rubber particles.

**Fig. 18:**
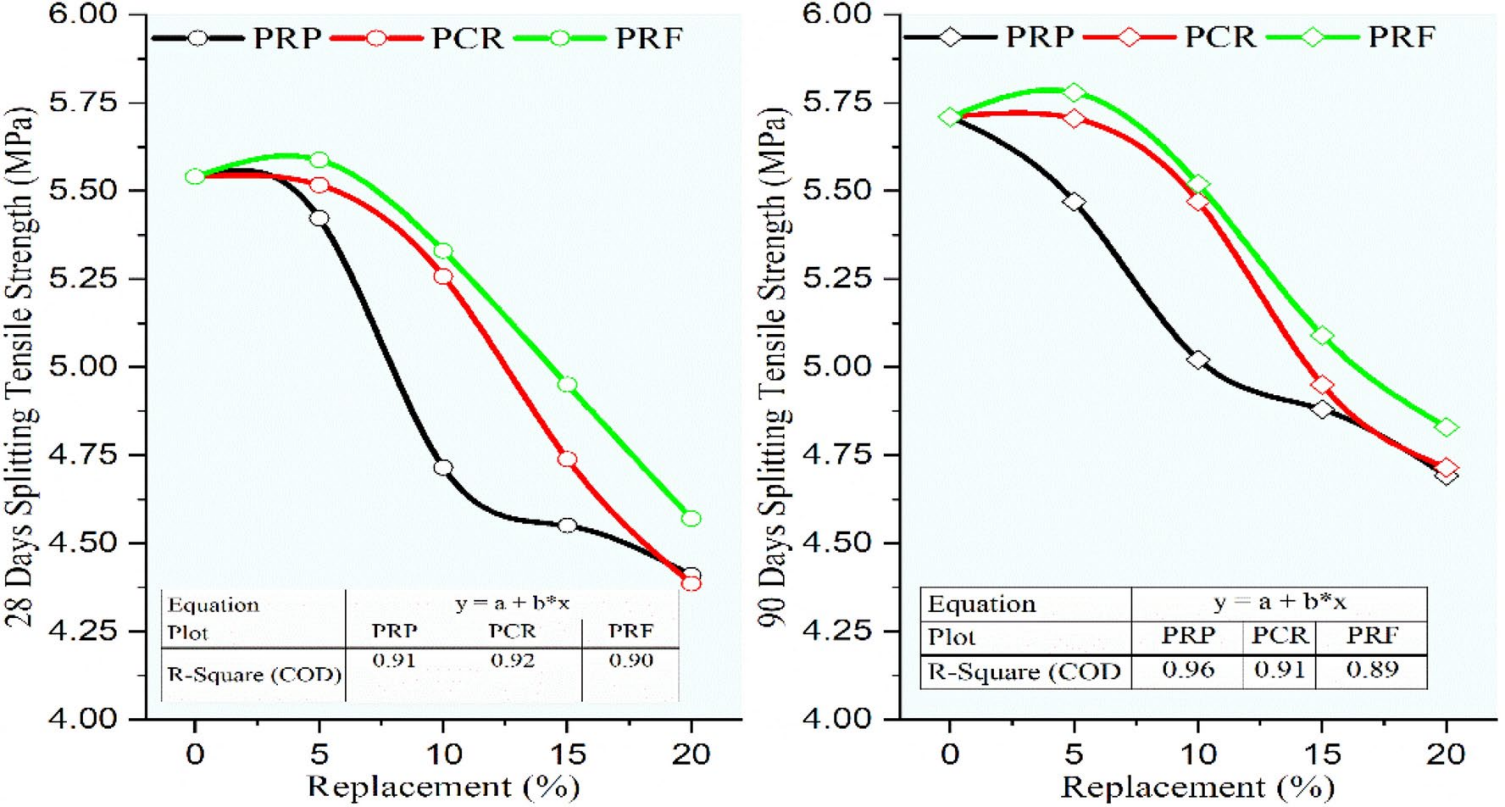
Variation in splitting tensile strength for control concrete and rc mixes with naoh pretreated rubber particles.

From Fig. 18, it was observed that the splitting tensile strength was improved for PRF5 at 28 and 90 days at 5.6 MPa and 5.8 MPa consequently. The further replacement in the rubber content decreased the strength, but again the decrement was less for RC mixes having RF and CR. Hence the coarser rubber particles performed better for splitting tensile strength. The RC mixes PRP20 and PCR20 showed almost the same decrease in strength at 20.43%, 17.77%, 20.85%, and 17.36% correspondingly for 28 and 90 days. Due to the pretreatment of rubber particles, the reduction in strength was lessened.

**Fig. 19:**
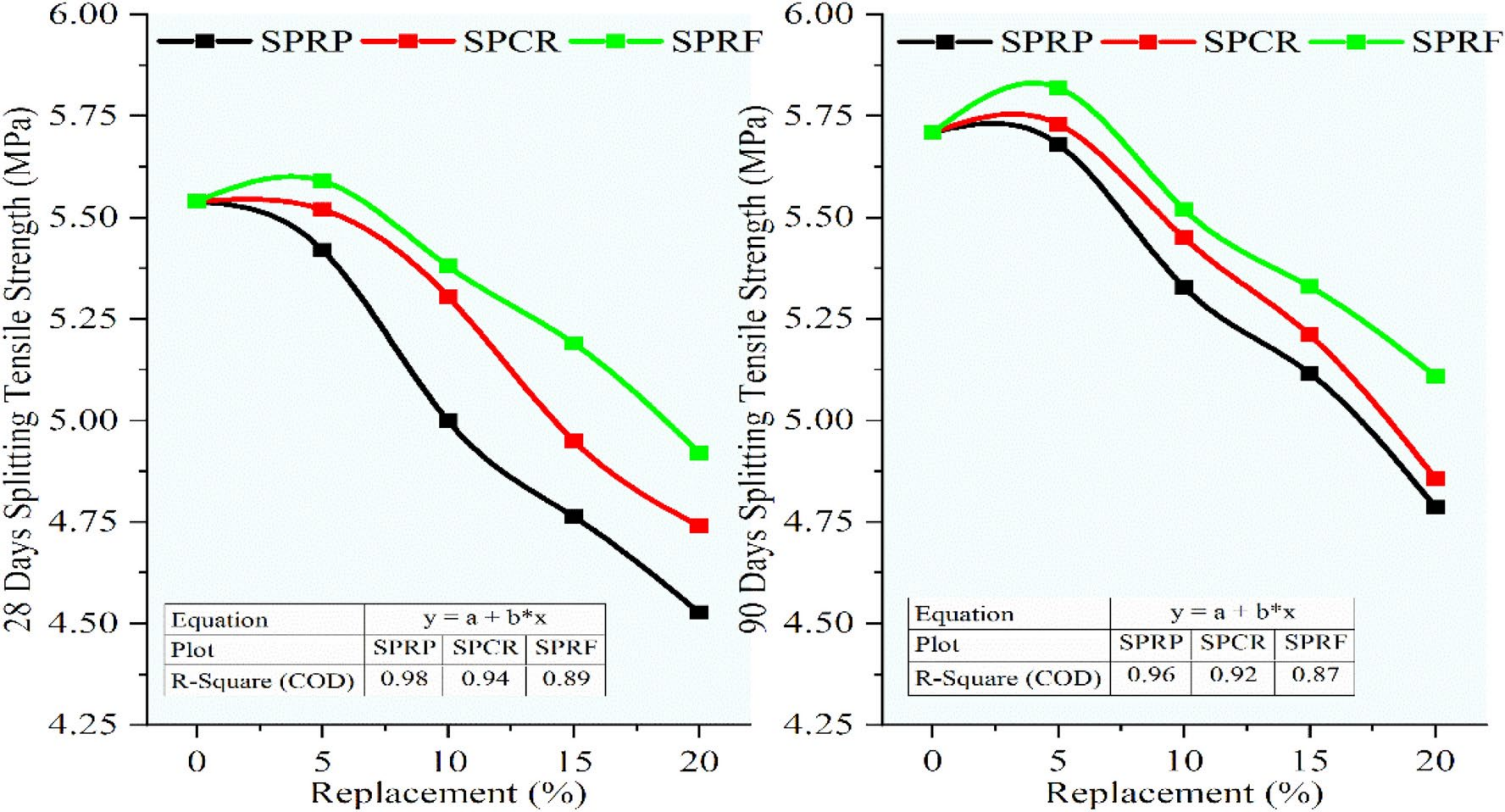
Variation in splitting tensile strength for control concrete and RC mixes with NaOH + silica fume pretreated rubber particle.

Figure 19 demonstrates the variation in splitting tensile strength for the control concrete and RC mixes having rubber particles pretreated with NaOH + silica fume. the strength was decreased with an increment in the rubber content while the amount of reduction was less as associated to the results obtained for RC mixes having rubber particles having no pretreatment and having pretreatment with NaOH. The RC mixes SPRF5 and SPRF10 exhibited strengths of 5.82 MPa, and 5.52 MPa at 90 days while further substitution showed a decrease in strengths by 6.61% and 10.38% at 90 days correspondingly for SPRF15 and SPRF20 mixes. The decrease in strength for RC mixes having RF at 20% was reduced from 17.36% to 10.38%. Authors^52^ recorded an increment in splitting tensile strength for concrete containing pretreated CR used as a part substitution of sand. From the study, it was confirmed that the finer rubber particles affect the strength more adversely than the coarser ones.

### Abrasion resistance

**Fig. 20:**
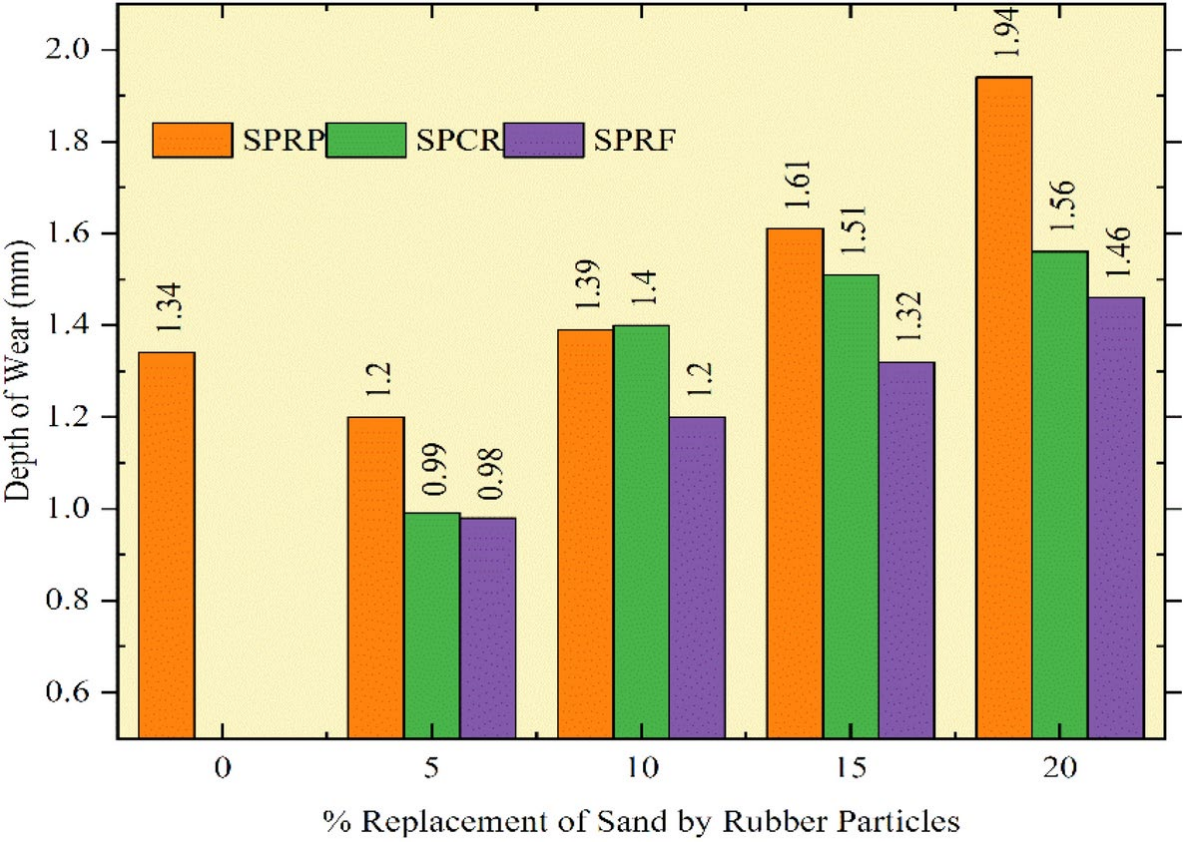
Abrasion resistance of concrete mixes with pretreated rubber particles.

Sliding of objects on one another, and rubbing causes the weakening of concrete, which is in turn considered as abrasion resistance of concrete. Abrasion resistance is measured by following norms set by^36^. The concrete tiles for all mixes are prepared as per specification and were tested on an Abrasion testing machine, rotation disc was kept at a speed of 30 revolutions/ minute. Referring the Fig. 20, the depth of wear for the control mix is found at 1.34 mm whereas the amalgamation of rubber particles rises the depth of wear for all concrete mixes. SPRF5 mix has given less depth as associated with the control mix, as the depth for SPRF5 was measured as 0.98 mm. The depth of wear for SPRF 10 concrete is found to be decreased by 10.4% and SPRF15 concrete is nearly equal to the depth for control concrete. For all concrete mixes with rubber particle sizes S-1, S-2, and S-3 the depth of wear is within the allowable limits specified by standards^36^, as it is suggested that the wear depth must not surpass 2 mm for heavy loads. Figure 16 displays the abrasion resistance for concrete mixes with pre-treatment of NaOH and silica fume mentioned in this research in the form of depth of wear. From the fallouts, it is observed that the modified RC with 10% replacement of sand by pretreated rubber can be used for substantial applications, where more abrasion of concrete is expected. Authors^46,53^, concluded from their research on RC for abrasion that the abrasion resistance is increased.

### Dynamic elastic modulus

**Fig. 21:**
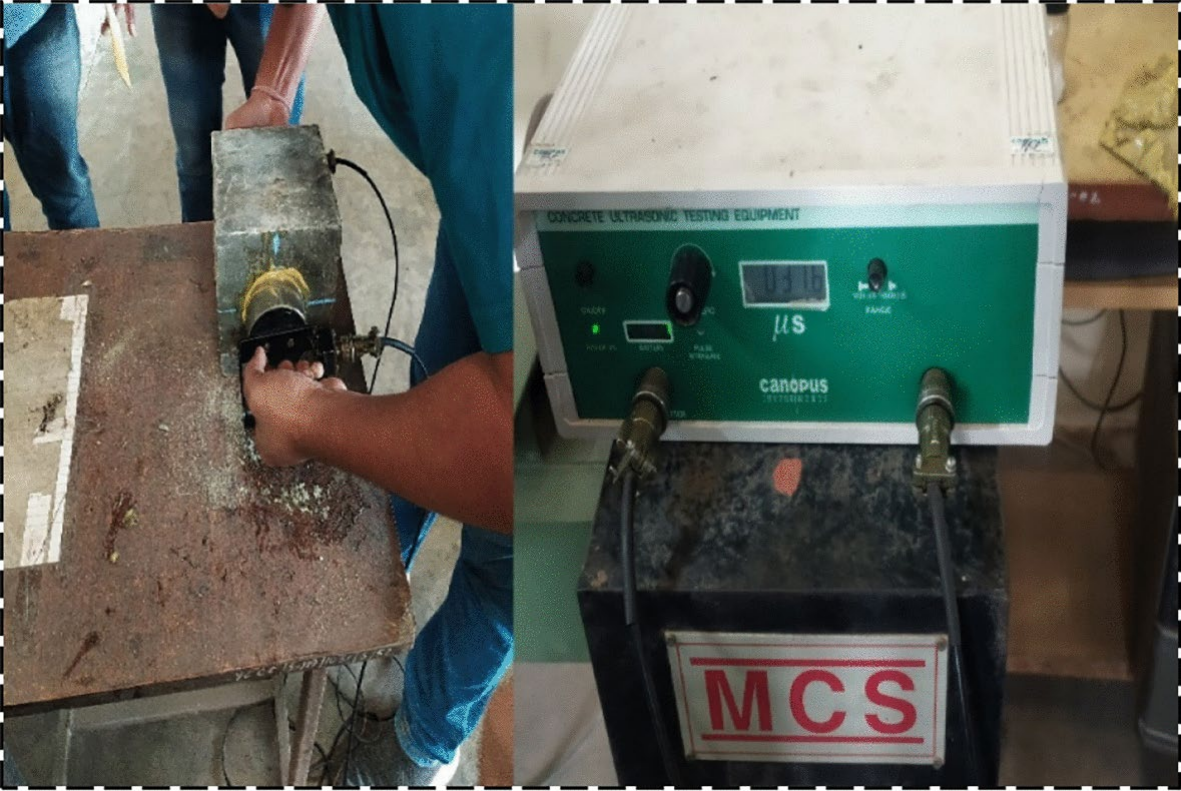
UPV testing on concrete cubes.

**Fig. 22:**
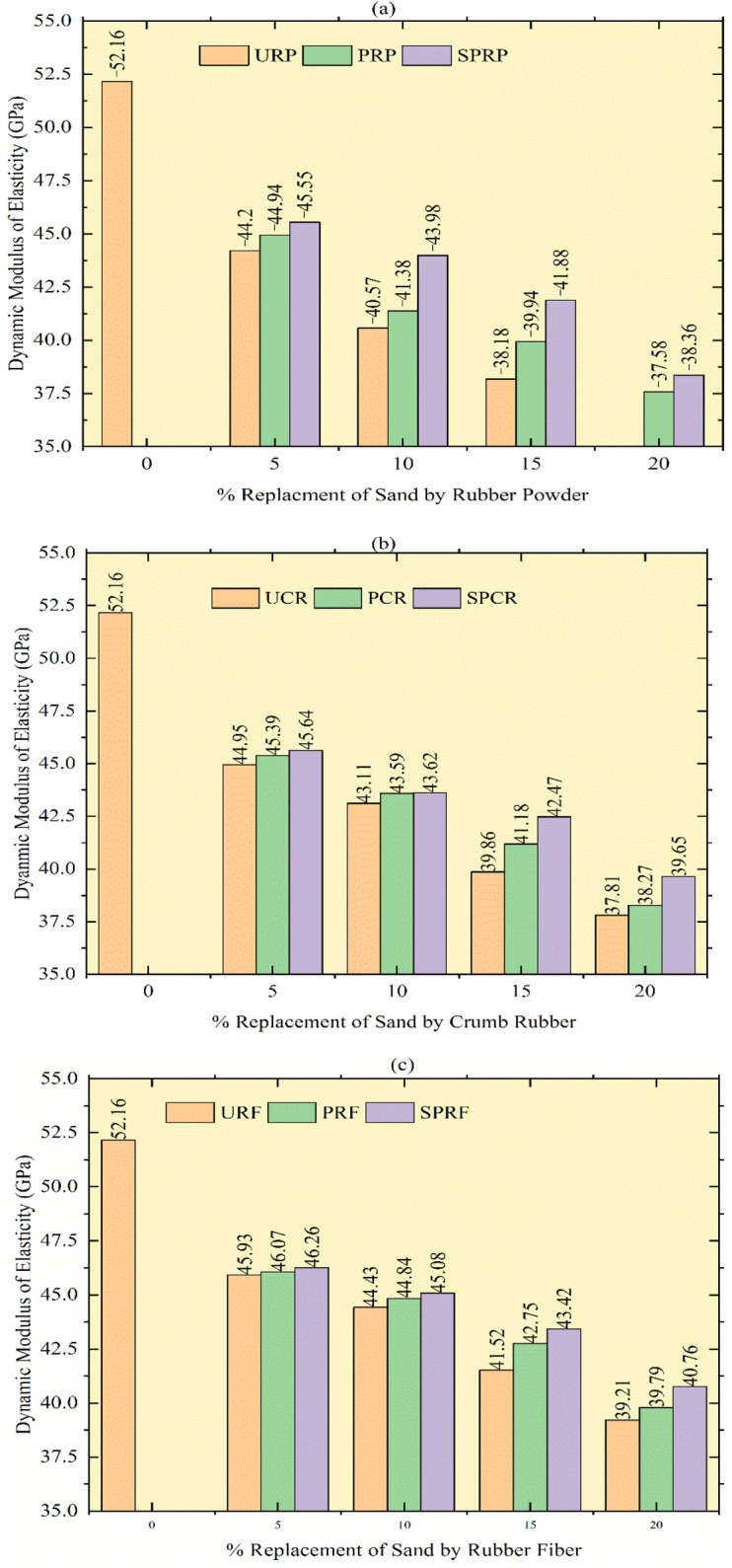
Dynamic Modulus of Elasticity: (**a**) Replacement of Sand by Rubber Powder, (**b**) Replacement of Sand by Crumb Rubber, and (**c**) Replacement of Sand by Rubber Fiber.

The impact of rubber particles on the dynamic elastic modulus (ED) was noted, and it was recorded that the insertion of rubber particles lessens the dynamic modulus of elasticity to a considerable amount. The moduli of elasticity declined with an upsurge in rubber content. The testing of UPV was demonstrated in Fig. 21 and the graphical representation of reduction in ED is shown in Fig. 22, for sand replacement by URP, UCR, and URF at 20% substitution, the decrease of 33.9%, 27.5%, and 24.8% correspondingly, when associated with control concrete. While other mixes with the utilization of treated rubber PRP, PCR, and PRF the reduction in ED was 27.9%, 26.6%, and 23.7% respectively for 20% replacement, and SPRP, SPCR, and SPRF have ED reduced by 26.4%, 24%, and 21.8% respectively for 20% substitution of sand by rubber particles. Researchers^46,54^ have recorded decrements in ED after the amalgamation of rubber with concrete. The finer rubber particles have more rate of decrement as compared to coarser ones and with control concrete. Along with this, the pretreatment of rubber particles diminished the drop ratio in contrast to mixes with untreated rubber particles.

### Water absorption of concrete mixes

As the RC is tested and compared for mechanical properties by performing the compressive, flexural, split tensile, and cylindrical compressive strength, it was discovered that the rubber particles employed in this investigation, which were rougher in texture, performed better. The different pretreatment methods adopted in the study have also contributed to imparting the enhancement of the mechanical strengths of RC mixes. The pretreatment method adopted in this study used immersion in 1 M NaOH solution, and the application of silica fume provided better results than other pretreatment methods. Along with improving the mechanical strengths of RC mixes the durability of modified concrete is needed to be checked for its life under different exposure conditions. When considering the longevity of concrete, the material’s ability to absorb water is crucial. This section of the study examined and compared the water absorption of RC mixes and control concrete for M60 grade concrete. Concrete’s water absorption was calculated using the BS 1881 standard^51^.

**Fig. 23:**
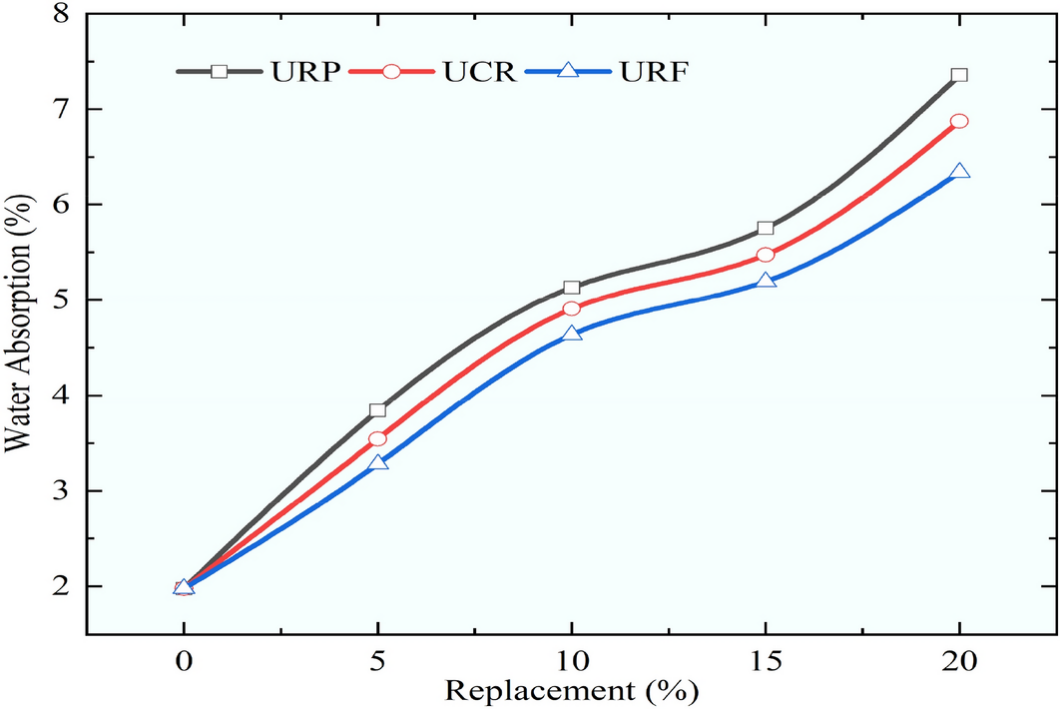
Variation in water absorption for control concrete and RC mixes with untreated rubber particle.

**Fig. 24:**
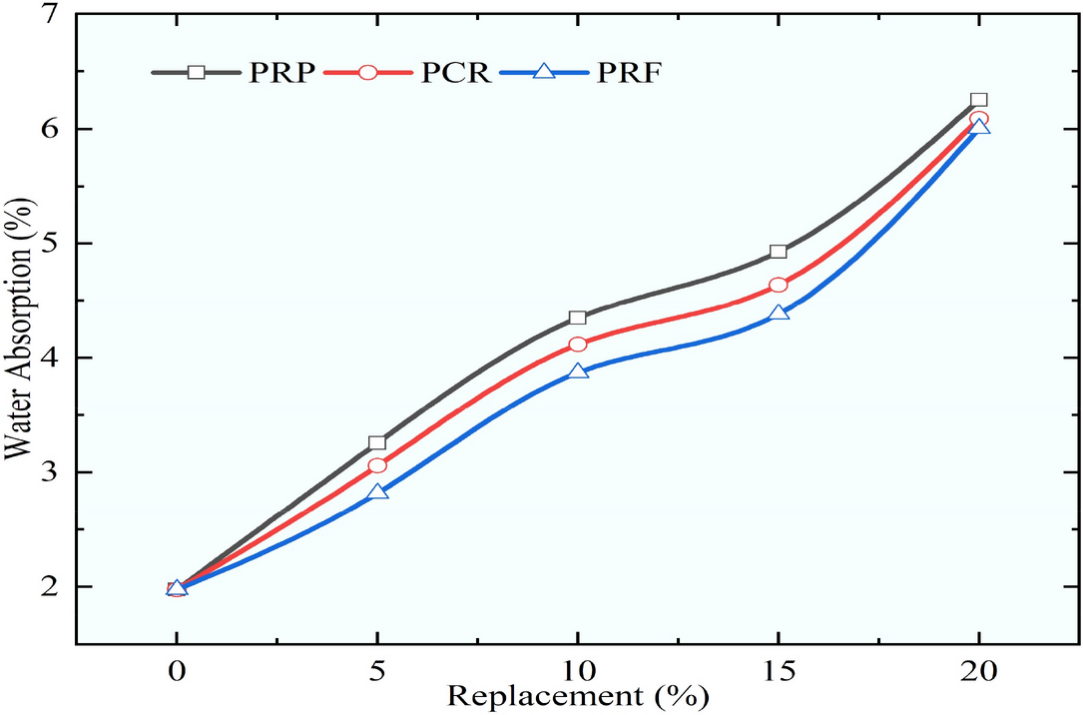
Variation in water absorption for control concrete and RC mixes with naoh pretreated rubber particles.

**Fig. 25:**
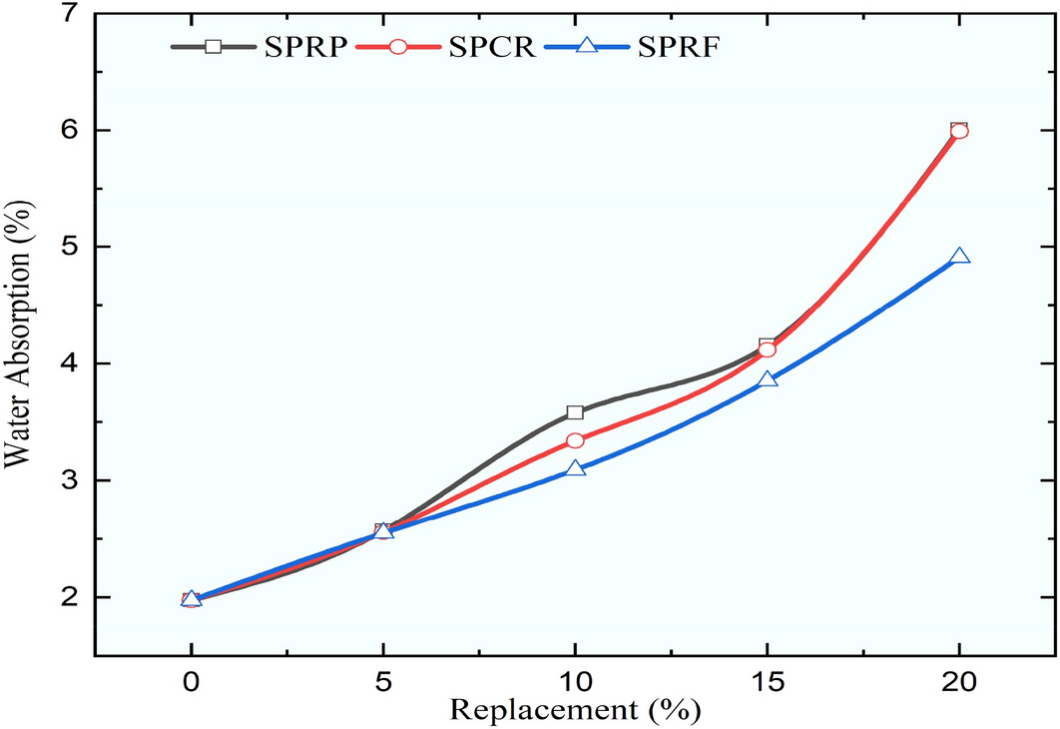
Variation in water absorption for control concrete and RC mixes with naoh + silica fume pretreated rubber particle.

Every mix that had more rubber in it had more water absorption for RC mixes. The findings of the water absorption test revealed a similar trend of rising percentage of water absorption regardless of the size of rubber particles. The results of water absorption are demonstrated in Figs. 23, 24, and 25 for different sizes of rubber particles and with different pretreatment methods. The maximum water absorption was noticed for the mix URP20 as it increased by 2.72 times when compared to the control concrete. The water absorption for control concrete was obtained as 1.98%. The mixes URP20, PRP20, and SPRP20 showed water absorption at 7.36%, 6.25%, and 6.01%. while the mixes UCR20, PCR20, and SPCR20 the water absorption was observed as 6.87%, 6.09%, and 5.99%. The mixes URF20, PRF20, and SPRF20 showed water absorption as 6.34%, 6.01%, and 4.91% respectively. From the results, it was observed that the water absorption was reduced by providing the pretreatment to the rubber particles. The least water absorption within the RC mixes was obtained for the RC mix SPRF5 as 2.55%, which was found to be the nearer value in comparison to the water absorption of control concrete. For all the RC mixes with a 5% substitution of sand with pretreated rubber particles the water absorption was found to be less than 3.5%.

### Microstructural analysis of rubber particles

**Fig. 26:**
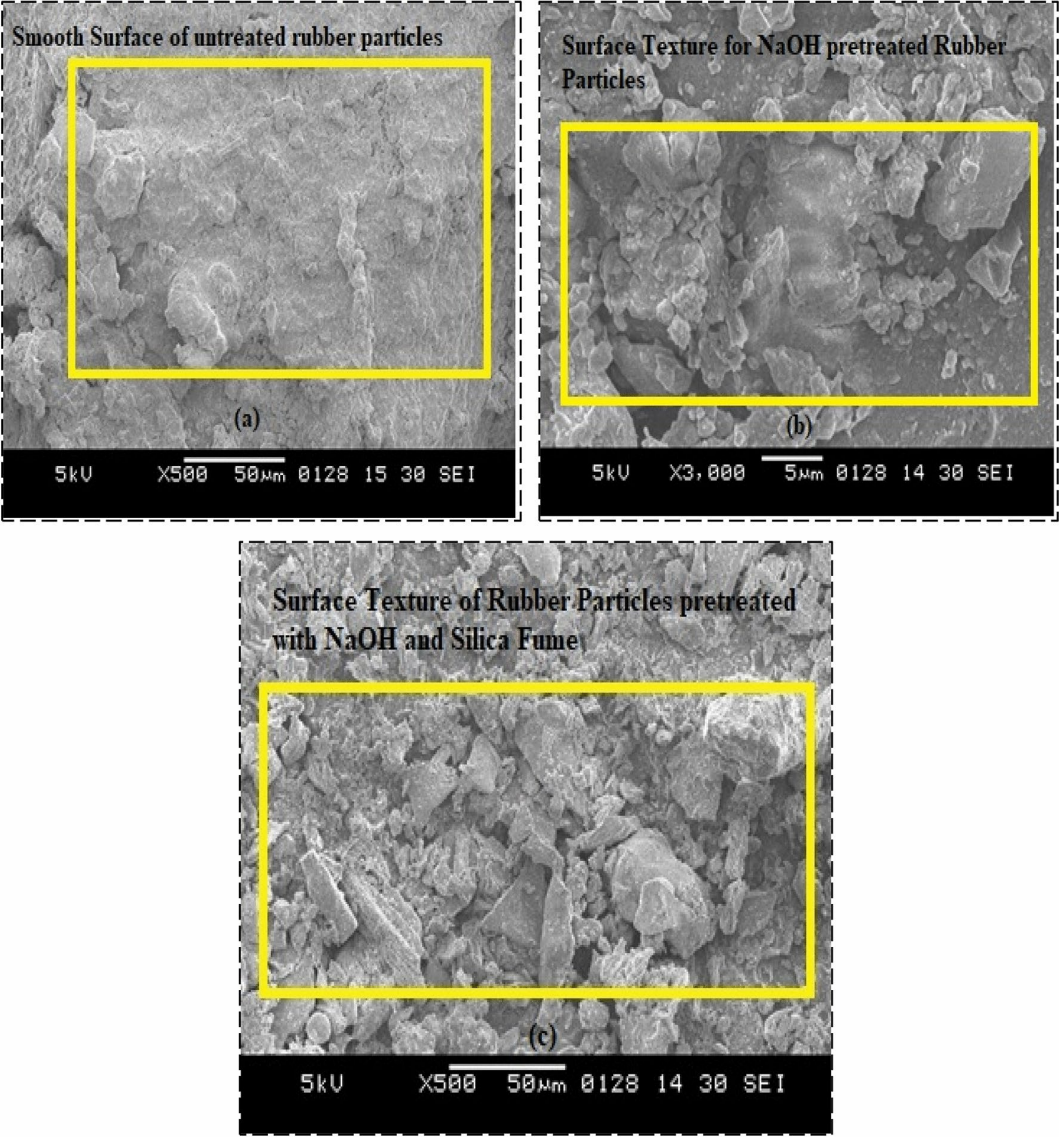
Microstructural Analysis of (**a**) Untreated Rubber Particles, (**b**) Rubber Particles Pretreated with NaOH, (**c**) Rubber particles pretreated with NaOH and Silica Fume.

From the results obtained for different tests performed on the hardened concrete, it is perceived that the functioning of the RC with pretreated rubber particles is enhanced in comparison to the RC with untreated rubber particles. Therefore, it can be commented that the pretreatment helped to boost the interfacial bonding of cement paste with rubber particles. Microstructural analysis of untreated and pretreated rubber particles is performed using Scanning Electron Microscopy (SEM) analysis. Figure 26a, b, and c demonstrates the SEM results for untreated rubber particles, rubber particles pretreated with NaOH, and rubber particles pretreated with NaOH and silica fume respectively. The roughness of the rubber particles was found to be improved and hence the pretreatment techniques may be useful to enhance the interfacial bonding in cement paste and rubber particles.

### Future recommendations

The use of industrial and polymer wastes in concrete as a partial substitution of constituents of concrete is a well-known practice, but the recycling of such concrete is still a grey area. The recycling of RC itself is challenging, and it can be recycled after its life cycle by using it as recycled aggregates. The use of discarded RC as landfilling can be a viable solution as it avoids direct rubber landfilling, which causes the leaching problem. Although these options are possible, this topic of recycling requires devising proper recycling strategies.

A thorough study is conducted in this research on the particle size variation of rubber particles when utilized as the fractional substitution of sand. As per the obtained results, it is seen that the concrete with finer rubber particles has a higher declination rate so, it is recommended that the blending of different sizes of rubber particles by shredding and sieving, in turns result in similar distribution curve as that of sand.

Although a detailed cost analysis was beyond the scope of this study, the economic feasibility could vary depending on factors such as the scale of production, the availability and cost of untreated waste rubber, and advancements in cost-effective pretreatment technologies. Additionally, the long-term benefits, such as reduced landfill waste and enhanced durability of rubberized concrete, could offset initial pretreatment costs in certain contexts. It will be the better opportunity to civil engineering industry to work on life-cycle and cost analysis for utilization of rubber waste in concrete.

## Conclusions

This investigational research was taken out to check the best suitable size of rubber particles and the influence of pre-treatment of rubber particles before their incorporation into the concrete as fractional substitution of fine aggregates. Reviewing the results and discussions section, this study has conclusions as follows.

- The workability of concrete is seen to be lessened with an upsurge in rubber content. More reduction in workability is noted for RC mixes with RP. In addition to this, the RC mixes with untreated rubber particles have shown more reduction than RC mixes with pretreated rubber particles. The maximum decline
- The density is found to be reduced by incorporating rubber particles into concrete. The reduction rate is more for the finer rubber particles. The pretreatment techniques adopted for the RC mix in the study showed higher densities than the RC mixes with untreated rubber particles, but the density for all RC mixes is less than the control concrete.
- The 28 and 90 days compressive strength for untreated rubber particles is found to be less than the control concrete. The maximum decrease in the compressive strength at 37.31% and 34.92% for 28 and 90 days is noted for the URP20 mix. The pretreatment methods adopted in the study showed an advancement in compressive strength. The RC mixes with CR and RF pretreated by NaOH and showed compressive strength almost identical to the characteristic compressive strength of M-60 concrete i.e. 60 MPa. The pretreatment method invented in the study showed promising results up to 10% substitution for RFs. The further replacement of sand beyond 10% revealed a reduction in the strength due to weak bonding among cement paste and rubber particles.
- The cylindrical compressive strength is determined to check the deformation ability of RC mixes and the control concrete. The cylindrical compressive strength for the RC mixes with untreated rubber particles and for rubber particles pretreated with NaOH is decreased with an increase in the rubber content. While the RC mixes with rubber particles pretreated with NaOH + silica fume showed almost the same strength for 10% replacement as that of control concrete at 90 days.
- The flexural strength test for M-60 grade concrete is assessed and compared according to the pretreatment method adopted. The flexural strength is linearly decreased with an increase in the untreated rubber particles. The maximum loss in flexural strength is observed at 20% substitution of sand by RP. The mixes URP5, UCR5, and URF5 at 28 days, and URP10, UCR10, and URF10 at 90 days have shown strength more than the least required flexural strength for M-60 concrete. The flexural strength of rubber particles pretreated with NaOH exhibited almost the unchanged strength as that of control concrete for the mixes PRP5, PCR5, and PRF10. The RC mixes pretreated with NaOH + silica fume showed the most promising results by reducing the reduction ratio by 55% when associated with the RC mixes with untreated rubber particles.
- The splitting tensile strength is less for RC mixes having untreated rubber particles than the RC mixes with pretreated rubber particles. The PRF5 mix showed strength superior than the control concrete. The mixes SPRF5 SPRF10 also obtained a strength higher than the control concrete at 28 and 90 days. The further addition of RFs in concrete decreased the strength by 6.61% and 10.38% respectively for 15% and 20% exchange with sand at 90 days.
- Abrasion resistance was recorded from the depth of wear of concrete tiles for different concrete mixes and detected that the depth of wear increased for concrete mixes with pretreatment in comparison to the control mix except for SPCR5 and SPRF5, but all depths are within the acceptable limits.
- The dynamic modulus of elasticity of the RC mixes is greatly reduced as compared to the control concrete. The reduction in dynamic elastic modulus enhances the flexibility of concrete, thus the RC mixes can be a viable alternative for conventional concrete to be used for the construction of structures in earthquake-prone areas, runways, shock-absorbing machinery pads, highway constructions, and crash barriers. The extreme lessening is perceived for the URP20 mix at 33.9% when contrasted to the control concrete. While for the RC mixes SPRF5 and SPRF 10 showed better performance in all other mechanical properties having the dynamic elastic modulus of 46.26 GPa and 45.08 GPa respectively in connection to the 52.19 GPa for the control concrete.
- The water absorption for all RC mixes is increased linearly with a surge in rubber content. The rate of increment varies with the size of the rubber particles and the pretreatment method. The less increase in water absorption is noted for the RC mixes pretreated with NaOH + silica fume which is the newly invented technique in this study. The minimum increase in water absorption at 20% substitution is noted for the SPRF mix as 4.91%.

Mixing silica fume with rubber particles treated using NaOH before dry mixing of concrete ingredients has shown better performance of RC, as properties of concrete are enhanced. According to the methodology adopted for this study and by comparing the results of mechanical tests for RC mixes with different sizes of rubber particles, up to 10% swap of sand by pretreated rubber fiber can be a feasible combination to introduce modified concrete using automobile waste and it can be a viable solution to make concrete sustainable and to avoid environmental hazards caused by stockpiling discarded tires. 

## Data Availability

The datasets used and/or analysed during the current study available from the corresponding author on reasonable request.

## Abbreviations

ELT: End-of-life tires
RC: Rubberized concrete
w/c: Water-cement ratio
NaOH: Sodium hydroxide
URP: Untreated rubber powder
UCR: Untreated crumb rubber
URF: Untreated rubber fibers
PRP: Pre-treated rubber powder
PCR: Pre-treated crumb rubber
PRF: Pre-treated rubber fibers
SPRP: Pretreated with silica fume and NaOH rubber powder
SPCR: Pretreated with silica fume and NaOH crumb rubber
SPRF: Pretreated with silica fume and NaOH rubber fibers
ASTM: American Society for Testing and Materials
IS: Indian standards
OPC: Ordinary portland cement
HRWR: High range water reducer
w/cm ratio: Water and cementitious powder ratio
UPV: Ultrasonic pulse velocity
SF: Silica fume
CR: Crumb rubber
RF: Rubber fiber
